# Time-resolved dual RNA-seq reveals extensive rewiring of lung epithelial and pneumococcal transcriptomes during early infection

**DOI:** 10.1186/s13059-016-1054-5

**Published:** 2016-09-27

**Authors:** Rieza Aprianto, Jelle Slager, Siger Holsappel, Jan-Willem Veening

**Affiliations:** 1 Molecular Genetics Group, Groningen Biomolecular Sciences and Biotechnology Institute, Centre for Synthetic Biology, University of Groningen, Nijenborgh 7, 9747 AG Groningen, The Netherlands; 2 Department of Fundamental Microbiology, Faculty of Biology and Medicine, University of Lausanne, Biophore Building, CH-1015 Lausanne, Switzerland

**Keywords:** *Streptococcus pneumoniae*, Transcriptomics, Dual RNA-seq, Host-microbe interaction, Adherence, Lung epithelial cells, Competence, Systems biology

## Abstract

**Background:**

*Streptococcus pneumoniae*, the pneumococcus, is the main etiological agent of pneumonia. Pneumococcal infection is initiated by bacterial adherence to lung epithelial cells. The exact transcriptional changes occurring in both host and microbe during infection are unknown. Here, we developed a time-resolved infection model of human lung alveolar epithelial cells by *S. pneumoniae* and assess the resulting transcriptome changes in both organisms simultaneously by using dual RNA-seq.

**Results:**

Functional analysis of the time-resolved dual RNA-seq data identifies several features of pneumococcal infection. For instance, we show that the glutathione-dependent reactive oxygen detoxification pathway in epithelial cells is activated by reactive oxygen species produced by *S. pneumoniae*. Addition of the antioxidant resveratrol during infection abates this response. At the same time, pneumococci activate the competence regulon during co-incubation with lung epithelial cells. By comparing transcriptional changes between wild-type encapsulated and mutant unencapsulated pneumococci, we demonstrate that adherent pneumococci, but not free-floating bacteria, repress innate immune responses in epithelial cells including expression of the chemokine IL-8 and the production of antimicrobial peptides. We also show that pneumococci activate several sugar transporters in response to adherence to epithelial cells and demonstrate that this activation depends on host-derived mucins.

**Conclusions:**

We provide a dual-transcriptomics overview of early pneumococcal infection in a time-resolved manner, providing new insights into host-microbe interactions. To allow easy access to the data by the community, a web-based platform was developed (http://dualrnaseq.molgenrug.nl). Further database exploration may expand our understanding of epithelial–pneumococcal interaction, leading to novel antimicrobial strategies.

**Electronic supplementary material:**

The online version of this article (doi:10.1186/s13059-016-1054-5) contains supplementary material, which is available to authorized users.

## Background

Lower respiratory tract infections (LRTIs), or pneumonia, claim more lives than any other communicable disease worldwide; the main etiologic agent behind this infection is the Gram-positive opportunistic pathogen *Streptococcus pneumoniae* (pneumococcus) [1]. Normally part of the human nasopharyngeal microflora, *S. pneumoniae* can invade the lower airways where it provokes host inflammatory and immune responses [2]. At the earliest stage of infection, pneumococcus adheres to epithelial cells and interacts intimately with the epithelium [3]. Meanwhile, host and microbe cross-communicate and simultaneously affect each other in a disruptive manner [2, 4]. This interspecies interaction activates numerous processes in epithelial and pneumococcal cells [5, 6]. To obtain comprehensive and meaningful biological knowledge of the infection processes involved in pathogenesis, simultaneous monitoring of the transcriptome changes in both species is required [7].

Lung epithelial cells perform vital roles during infection. First, the cells form a physical barrier to the external environment. On top of these cells, a thick layer of epithelium-derived mucus offers extra protection that traps and removes pathogens [8]. Mucins, the main component of mucus, are large glycoprotein polymers rich in sialic acids and other aminosaccharides [9]. Additionally, epithelial cells kill pathogens directly by producing antimicrobial peptides, e.g., defensins and cathelicidins [10]. Moreover, epithelial cells regulate innate immune responses by secreting a wide array of pro-inflammatory cytokines that recruit neutrophils and activate macrophages [11]. Finally, epithelial cells activate adaptive immune cells, including dendritic cells and T cells, via chemokine expression [12].

Pneumococcal adherence to epithelial cells is the first necessary step to pathogenesis [13]. In order to adhere, pneumococcus must quickly shed the thick exopolysaccharide capsule, which protects against phagocytes [14, 15]. The shedding exposes surface adhesion factors and desensitizes the bacterium from antimicrobial peptides [16, 17]. Subsequently, *S. pneumoniae* must acquire nutrients to support growth and, at the same time, evade host immune responses [18]. Pneumococcal factors may be involved in multiple processes; e.g., PsaA, a surface-exposed protein, acts concurrently as adhesion factor and manganese transporter [19]. The scarce manganese [20] helps in neutralizing reactive oxygen species (ROS) and in bacterial fitness [21].

Interspecies interaction during infection is a complex process which necessitates rapid and massive adaptation for epithelial and pneumococcal survival. During the adaptation, transcriptional changes are a focal point, in both the host [22] and pathogen [23]. RNA-sequencing (RNA-seq) delivers genome-wide quantitative snapshots of the transcriptome [24]. In a thought experiment, Vogel and co-workers argued that simultaneous profiling of host and pathogen transcriptomics by dual RNA-seq might provide valuable insights for infection biology [7]. Recent dual RNA-seq studies were successful in elucidating the host–pathogen regulatory network in *Candida albicans* [25], the role of small RNAs (sRNAs) in the intracellular pathogen *Salmonella typhimurium* [26], cross-talk in the Gram-negative LRTI pathogen *Haemophilus influenzae* [27], and transcription profiles in the protozoan *Leishmania major* [28] during infection.

Here, we exploited the dual RNA-seq approach to simultaneously monitor the transcriptome cross-talk between lung alveolar epithelial cells and pneumococci during early infection. Due to the transient and highly dynamic nature of the transcriptome [29], we monitored the transcriptional changes in a time-resolved manner. Moreover, since pneumococcal adherence to epithelial cells determines the outcome of early infection, we compared a mostly adherent unencapsulated mutant and mostly non-adherent wild-type encapsulated *S. pneumoniae* strain to allow specific transcriptional interrogation on adherence. Additionally, we confirmed our dual RNA-seq gene expression data by quantitative real-time PCR (qRT-PCR) and quantitative fluorescence microscopy to visualize pneumococcal proteins and thereby confirm several novel biological observations identified in the dataset. Finally, we developed a user-friendly online database (http://dualrnaseq.molgenrug.nl), giving access to our detailed time-resolved dual transcriptome data to the pneumococcal, microbiology, immunology, and pulmonology research communities.

## Results

### Model of early pneumococcal infection of epithelial cells

**Fig. 1:**
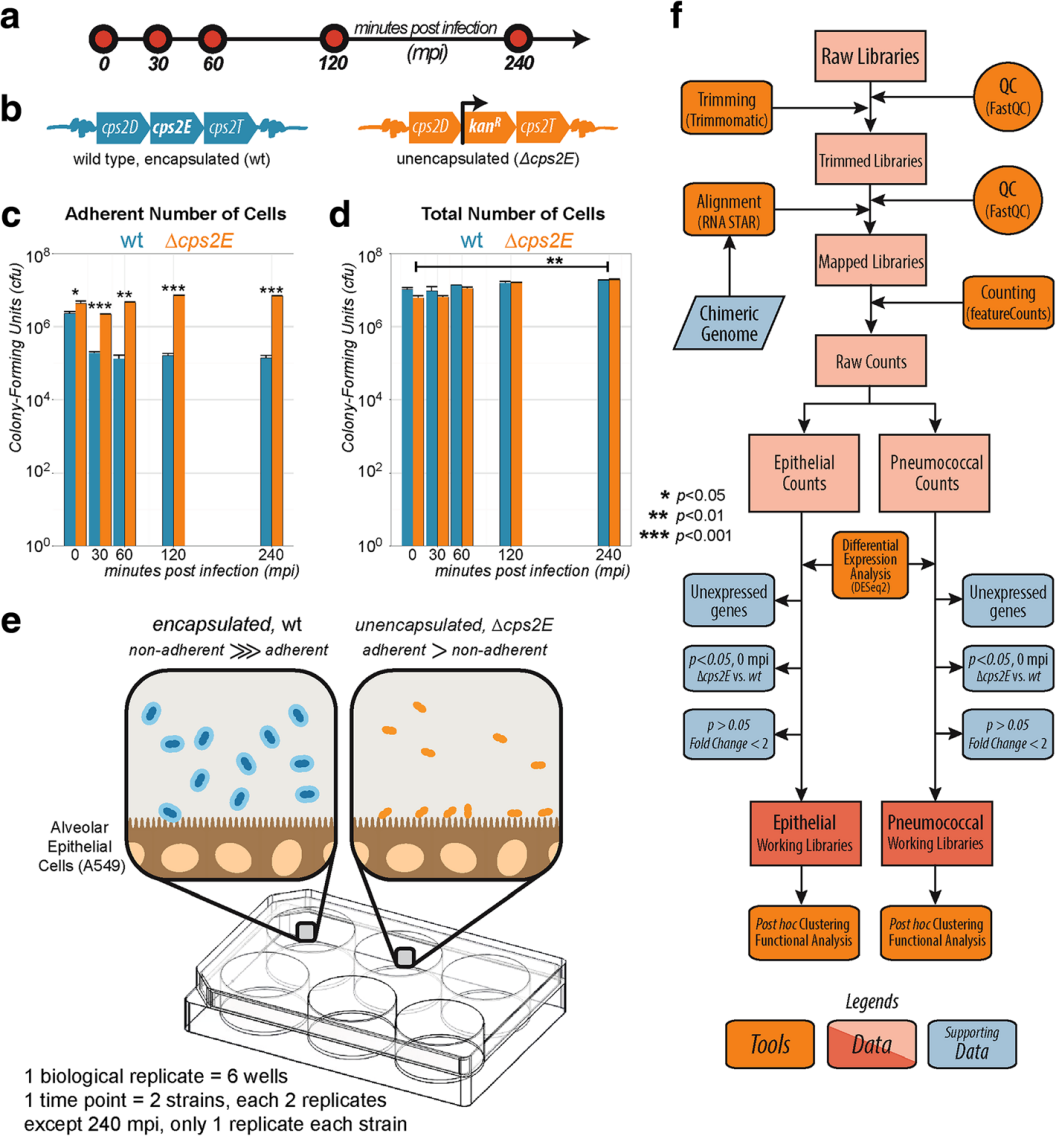
The early infection model. A confluent monolayer of alveolar epithelial cells (A549) was co-incubated with *S. pneumoniae* strain D39 at an MOI of 10 (ten pneumococci per epithelial cell). **a** Five infection time points were interrogated: 0, 30, 60, 120, and 240 minutes post-infection (*mpi*). **b** Since adherence is a hallmark of infection, we used an unencapsulated *S. pneumoniae* strain (*∆cps2E*), which adheres more readily to epithelial cells than its encapsulated parental strain. **c** At 30 mpi, *∆cps2E* (*orange bar*) showed significantly (*p* < 0.001) more adherent cells than the wild-type (*wt*) parental strain (*cyan bar*). Data are presented as mean ± standard error of the mean. **d** At 240 mpi, both strains multiplied significantly (*p* < 0.01) with no significant difference between them. **e** The setup with the encapsulated strain contains more free-floating than adherent bacteria while *∆cps2E* has a higher fraction of adherent bacteria. **f** After quality control (*QC*), low-quality reads were trimmed and aligned to a synthetic chimeric genome. Aligned reads were counted and classified as epithelial or pneumococcal counts. We removed three gene fractions and performed clustering and functional enrichment of the working libraries

During the first phase of LRTI, *S. pneumoniae* adheres to the sterile apical side of epithelial cells and replicates. At this point, both the bacterial transcriptome and the epithelial transcriptome change in response to the interaction [5, 6]. We aimed to recapitulate these events in an in vitro model consisting of co-incubation of the pathogenic *S. pneumoniae* strain D39 (serotype 2) and a confluent layer of type II lung alveolar human epithelial cells (A549) at a multiplicity of infection (MOI) of 10, i.e., ten pneumococci per epithelial cell (Fig. 1a). An MOI of 10 was used so that a minimum amount of pathogen per host cell was needed to provoke an interspecies transcriptional interaction, minimizing epithelial cell death within the first 240 minutes after co-incubation. Five time points up to 4 h after infection were selected to capture both early transcriptome responses (30 and 60 minutes post-infection (mpi)) and later responses (120 and 240 mpi). Longer co-incubation times were not probed as, starting at 6 h post-infection, epithelial cells rounded up and detached in our model. Six technical replicates (individual wells) were pooled into one biological replicate. Two biological replicates were used for each time point, except for 240 mpi, for which we obtained only one replicate (Fig. 1e). In order to elucidate adherence-specific expression, we incorporated an isogenic unencapsulated D39 strain (*∆cps2E*; Fig. 1b) with increased adherence to epithelial cells into the model [30]. The capsular mutant showed significantly greater capacity to adhere to epithelial cells than its encapsulated parental strain (*p* < 0.001; Fig. 1c). During infection, the total number of cells of both strains was significantly (*p* < 0.01) increased after 4 h (Fig. 1d), showing that both strains multiply in the model, thereby recapitulating one of the characteristics of infection. To minimize transcriptional changes because of sample handling, we did not separate cellular mixtures before total RNA isolation (epithelial cells, adherent pneumococci, and free-floating pneumococci; Fig. 1e; see “Methods”).

To analyze the time-resolved dual RNA-seq dataset, a combination of freely available bioinformatics tools was used (Fig. 1f). First, raw reads were trimmed [31] and aligned [32] to a chimeric genome containing the concatenated genome of *Homo sapiens* (Ensembl, release 84) and *S. pneumoniae* (Ensembl, release 31, bacteria 13 collection [33]). One-step mapping was chosen to minimize rates of false negatives. Reads were then separately counted [34] and classified as either epithelial or pneumococcal. Following differential gene expression analysis, three groups of genes were removed (see “Dual RNA-seq generates high-quality datasets with clusters of epithelial and pneumococcal co-expressed genes” below) and unbiased automatic clustering [35] and functional enrichment were performed [36, 37].

### Dual RNA-seq generates high-quality datasets with clusters of epithelial and pneumococcal co-expressed genes

**Fig. 2:**
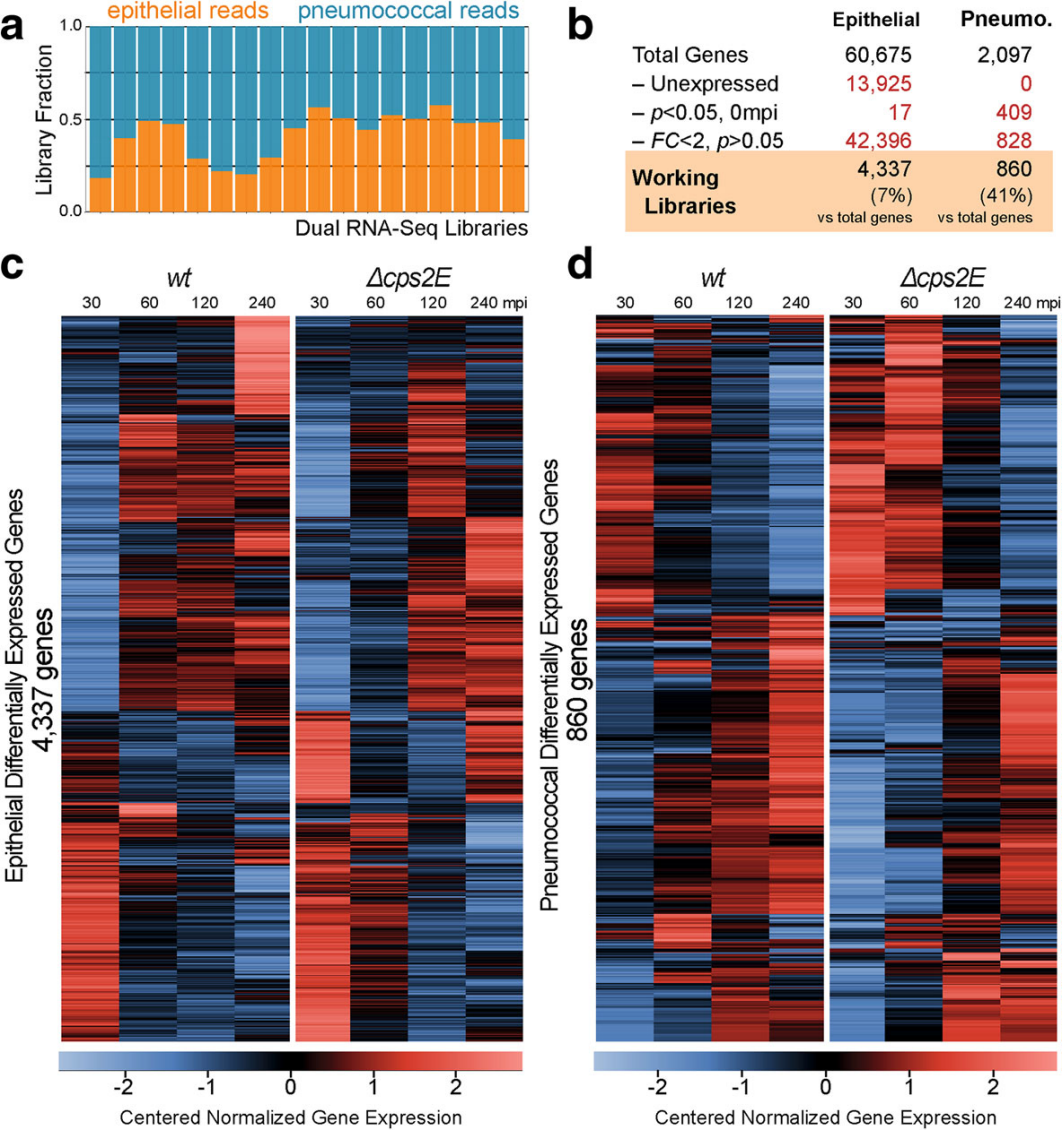
Dual RNA-seq generates high-quality datasets suitable for probing host–pathogen transcriptomes. **a** On average, there are 70 million reads per library: 42 % of the reads aligned to the human genome and 58 % to the *S. pneumoniae* D39 genome. **b** In order to simplify downstream analysis, we excluded three gene fractions: unexpressed genes, i.e., those without counts in any libraries; genes that were differentially expressed at 0 mpi (*p* < 0.05) between *∆cps2E* and wild-type (*wt*) libraries; and genes with no statistical significance (*p* > 0.05) and fold changes (FC) < 2 in all comparisons. After exclusion, the epithelial working libraries contained 4337 genes (7 % of all human genes) while the pneumococcal working libraries contained 860 genes (41 % of all pneumococcal genes). **c** Gene expression in epithelial working libraries was normalized, centered, and clustered. The *left panel* shows epithelial genes in response to the encapsulated strain while the *right panel* shows the epithelial response to ∆*cps2E S. pneumoniae* at different time points. Clear clusters of co-expressed epithelial genes can be observed in the heat map. *Blue* indicates relatively lower expression while *red* indicates a higher value. **d** Pneumococcal expression: the *left panel* shows the wild-type pneumococcal response to epithelial cells, while the *right panel* shows the response of the ∆*cps2E* strain

Dual RNA-seq generated single-end 75-nucleotide reads. We sequenced to such a depth that, on average, each library has 70 million reads (30 to 95 million). After adapter trimming and removal of low-quality reads, we retained, on average, 92.0 % (89.0–92.9 %). Subsequently, on average, 99.2 % of reads (98.9 to 99.6 %) successfully aligned to the chimeric genome. Additionally, we concluded that dual rRNA depletion was successful since only 0.003 % of human and 0.06 % of pneumococcal reads mapped and counted as ribosomal RNAs. On average, for each library, we counted 29 million epithelial reads (42 % of total counted reads) and 41 million pneumococcal reads (58 % of total counted reads) (Fig. 2a). The high number of reads in each library and the high fraction of usable reads highlight the quality and suitability of our approach for dual RNA-seq: simultaneous total RNA isolation, dual rRNA depletion, and cDNA library preparation. Furthermore, principal component analysis (PCA) showed no evidence for batch effects (Additional file 1: Figure S1). Relative enrichment of pneumococcal reads may stem from the total RNA isolation protocol (see “Methods”). Nevertheless, each library contained sufficient epithelial reads for differential gene expression analysis [38].

To simplify further analyses, we excluded three gene fractions (Fig. 2b). First, we removed unexpressed genes, i.e., those without any counts in all libraries (13,925 (23 %) epithelial genes and none of the pneumococcal genes). The relatively large fraction of unexpressed epithelial genes might be due to the relatively low sequence coverage (average coverage of 1.8). Nevertheless, it is in accordance with recent studies on the human epithelial transcriptome [39, 40]. Second, we excluded genes that were differentially expressed (*p* < 0.05, adjusted *p* value by DESeq2) at 0 mpi between unencapsulated (*∆cps2E*) and encapsulated (wild-type) libraries. While 17 epithelial genes were removed, 409 (20 %) pneumococcal genes were already differentially expressed at 0 mpi. Although a polar effect due to *cps2E* disruption can explain differential expression of genes in the 17-kb-long *cps* operon, it remains unknown why other genes were differentially expressed. We speculate that constructing the thick exopolysaccharide capsule requires specific transcriptional fine-tuning of numerous genes outside the *cps* locus. Finally, we removed genes with no significant difference (*p* > 0.05) and genes with fold changes (FC) less than 2 in all comparisons (Additional file 1: Figure S2). In total, the epithelial working libraries contained 4337 (7 % of total) genes and the pneumococcal working libraries 860 (41 % of total) genes.

To compare gene expression, we normalized expression values using DESeq2 [41] and centered and clustered the values [35]. Note that differential gene analysis was performed before excluding any of the gene fractions (Fig. 1f). The centered normalized values were visualized as heat maps, divided into two panels, one for each bacterial strain. Strikingly, heat maps showed obvious clusters of co-expressed genes and clear gene expression differences between adhering (*∆cps2E*) and less-adhering (wild type) bacteria (Fig. 2c, d). Specifically, the left panel of Fig. 2c shows the epithelial transcriptional response when exposed to encapsulated *S. pneumoniae* at different time points (30, 60, 120 and 240 mpi) while the right panel displays the response when in contact with the unencapsulated strain. Vice versa, co-expressed clusters of pneumococcal genes are differentially expressed when the bacteria were in contact with human epithelial cells (Fig. 2d).

Making raw data publicly available has been common practice in recent years, as we have done for this project (Gene Expression Omnibus (GEO) accession number GSE79595). Unfortunately, publicly available datasets are not directly explorable for the extraction of biological insights for the majority of researchers. Therefore, we built an easily accessible online platform which hosts the complete dual RNA-seq database (http://dualrnaseq.molgenrug.nl). To access and visualize the data, users can simply select the gene of interest (or multiple genes of interests) and examine their expression during early infection (Additional file 1: Figure S3). Expression data can be downloaded and opened in common spreadsheet software, e.g., Microsoft Excel®. To visualize expression, users can choose from three normalization methods: DESeq2 normalization [41], TPM (transcript per million [42], and log_2_-transformed TPM values.

### Validation of dual RNA-seq by qRT-PCR and pneumococcal protein fusions

**Fig. 3:**
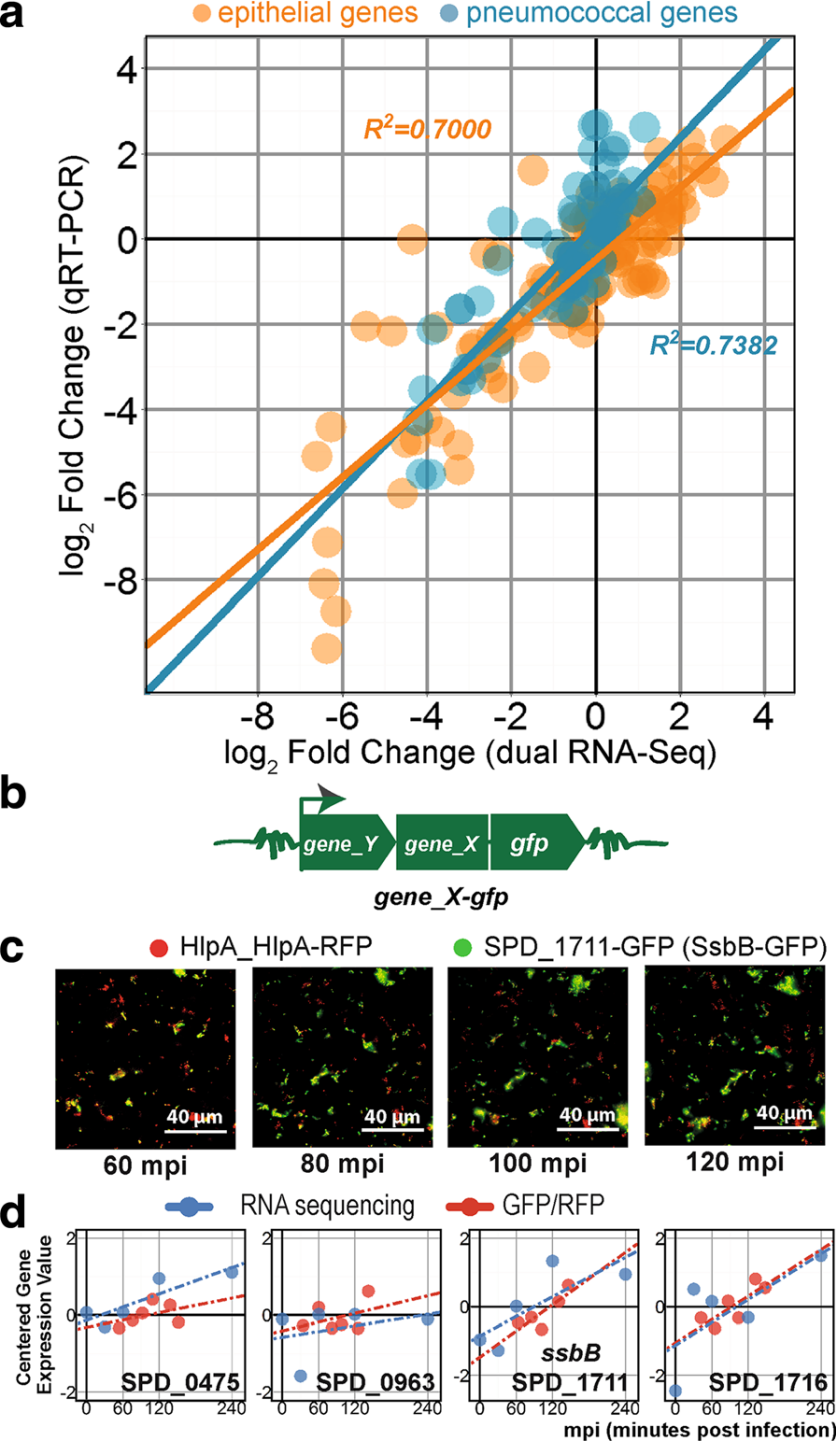
Validation of dual RNA-seq. **a** We confirmed dual RNA-seq gene expression values by qRT-PCR. The infection study was repeated in duplicates and total RNA was isolated as previously described. Ten human and 19 pneumococcal genes were chosen as validation targets. We plotted fold changes from qRT-PCR against dual RNA-seq fold changes and observed a high degree of correlation for both species (*R* ^*2*^ > 0.7, Pearson). **b** We also confirmed pneumococcal gene expression at the protein level by quantitative fluorescence microscopy. Four target genes (SPD_0475, SPD_0963, SPD_1711, and SPD_1716) were C-terminally tagged with green fluorescent protein (GFP) at their own locus. GFP fusions were introduced in the *∆cps2E* strain expressing red fluorescent protein (RFP) fused to HlpA. **c** Non-deconvolved image of SPD_1711*-*GFP up to 120 mpi. While RFP emitted a relatively constant signal, the GFP signal increased. **d** Dual RNA-seq expression values superimposed on the GFP/RFP ratio. To some extent, transcriptional changes corresponded to protein expression

To validate the dual RNA-seq data by qRT-PCR, we chose ten epithelial genes (*ABCC2*, *AKR1B10*, *AKR1C3*, *ALDH1A1*, *DEFB1*, *DKK1*, *IDH1*, *NOLC1*, *PTGES*, and *TXNRD1*) and 19 pneumococcal genes (*amiC*, *blpY*, *dinF*, *hrcA*, *infC*, *lytA*, *malC*, *manL*, *msmR*, *nrdD*, *pulA*, SPD_0249, SPD_0392, SPD_0475, SPD_0961, SPD_0990, SPD_1517, SPD_1711, and SPD_1798). The target genes were selected because of their varied expression profiles: increasing, decreasing, or unchanged. The cycle thresholds (Ct) for epithelial transcripts were normalized against *ACTB* (β-actin) while pneumococcal transcripts were normalized to *gyrA* (gyrase A). The reference genes were highly expressed and did not show significant changes (*p* > 0.05, FC < 2) between any time points during early infection. The qRT-PCR fold change was calculated by the ∆∆Ct method [43] to one time point. Fold changes obtained by qRT-PCR and dual RNA-seq showed a relatively high correlation for both species: *R* ^*2*^ = 0.70 for epithelial transcripts and *R* ^*2*^ = 0.74 for pneumococcal transcripts (Fig. 3a), validating the reliability of the dual RNA-seq data.

Since transcript levels do not necessarily correspond with protein expression [44, 45], we quantified four pneumococcal protein levels whose genes showed upregulation during adherence to epithelial cells. We fused a fast-folding variant of the green fluorescent protein (GFP) to the carboxyl termini of SPD_0475, SPD_0963, SPD_1711, and SPD_1716 at their own locus while preserving all upstream regulatory elements (Fig. 3b). We transformed these constructs into an unencapsulated strain constitutively expressing a red fluorescent protein (RFP) fused to a housekeeping gene (*hlpA_hlpA-rfp*) [30]. SPD_0475 encodes a 204 amino acid CAAX amino terminal protease with unknown function, SPD_0963 encodes a 45 amino acid hypothetical protein, SPD_1711 (132 amino acids) was described as a single-stranded DNA binding protein and may assist in competence [46], and SPD_1716 is a 63 amino acid ortholog of cell wall or choline - binding protein in other *Streptococcaceae*.

We imaged adherent *S. pneumoniae* with fluorescence microscopy during the indicated time points (Fig. 3c). Since (i) RFP serves as an accurate proxy for cell number and viability [30], (ii) *hlpA* does not change during early infection (*p* > 0.05, FC < 2), and (iii) the ratio between GFP and RFP indicates relative expression of the protein of interest (red circle and line, Fig. 3d), we were able to quantify the proteins of interest. Gene expression values from the dual RNA-seq data (blue circles and line, Fig. 3d) show a degree of correlation with protein level in three out of the four cases, suggesting that pneumococcal transcriptional changes reflect, to some extent, changes in protein level [47].

### Pneumococcal ROS induce expression of glutathione-mediated detoxification genes in epithelial cells

**Fig. 4:**
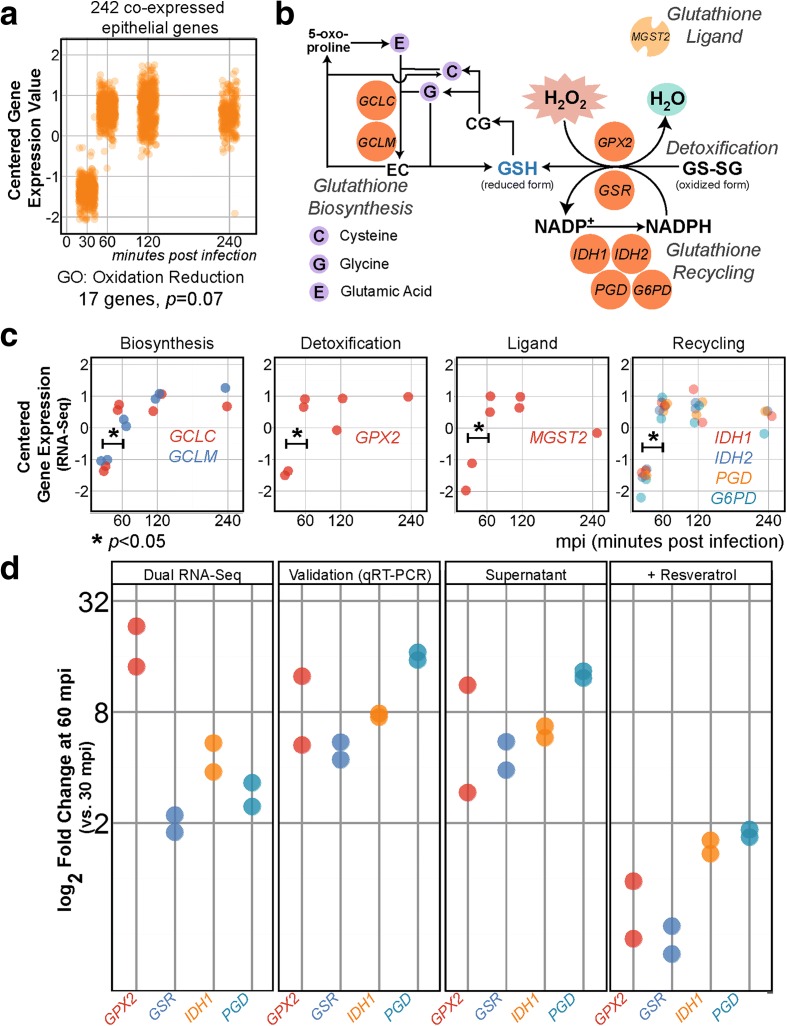
Epithelial glutathione-associated genes are activated in response to pneumococcal ROS. **a** We clustered epithelial working libraries exposed to wild-type pneumococci and found a cluster of 242 co-expressed genes showing sustained upregulation (*p* < 0.05, FC > 2) at 60 mpi compared with 30 mpi. Gene ontology (GO) analysis showed that “oxidation reduction” was enriched in 17 genes. **b** *GPX2*, encoding glutathione peroxidase-2, is one of the enriched genes. Eight genes are associated with glutathione (*GSH*), an important antioxidant. The main glutathione-associated processes are biosynthesis of glutathione and detoxification of ROS assisted by ligand and glutathione recycling. **c** Between 30 and 60 mpi, expression of *GCLC* increased 2.7 ± 1.1 times and of *GCLM* 2.3 ± 1.2 times. Expression of *GPX2*, the main detoxification gene, increased 18.1 ± 1.3 times while that of the gene encoding its ligand, *MGST2*, increased 3.1 ± 1.2 times. Genes involved in the recycling of glutathione were activated: *IDH1*, 4.5 ± 1.2; *IDH2*, 2.3 ± 1.2; *PGD*, 2.9 ± 1.2; and *G6PD*, 6.5 ± 1.2. **d** We validated *GPX2*, *GSR*, *IDH1*, and *PGD* expression using qRT-PCR. Epithelial incubation with pneumococcal supernatant showed similar upregulation of glutathione-associated genes. Addition of resveratrol (100 μM) into the model diminished the upregulation (FC < 2) altogether

Along with pneumococcal adherence and multiplication, we aimed to recapitulate the host response in our model. We hypothesized that the epithelial transcriptome adapts in response to bacterial presence, independent of adherence. To identify the responsive genes, we clustered epithelial working libraries exposed to wild-type pneumococci; 242 epithelial genes were co-expressed (Fig. 4a), i.e., lowly expressed at 30 mpi, then with sustained upregulation thereafter. Gene ontology (GO) enrichment [36] indicated that 17 of this subset are associated with oxidation and reduction (*p* = 6.9 × 10^−2^). *GPX2* (glutathione peroxidase-2), which catalyzes the oxidation of glutathione, is one of the genes in this subset.

Glutathione, a tripeptide of glutamic acid, cysteine, and glycine, is produced and secreted by epithelial cells [48]. This vital molecule is biosynthesized through amino acid polymerization and, in the presence of ROS (e.g., hydrogen peroxide, lipid superoxide, or oxygen radicals), it readily donates an electron or hydrogen atom to quench them. The process is assisted by ligands and glutathione peroxidase (GPX2). Oxidized glutathione can be recycled by glutathione reductase (GSR) dependent on NADPH (Fig. 4b). Alternatively, glutathione conjugates and neutralizes ROS [49]. Expression of eight glutathione-associated genes showed a sustained significant increase in epithelial cells exposed to the encapsulated strain (*p* < 0.05, 60 versus 30 mpi; Fig. 4c).

To validate the abovementioned finding, we repeated the experiment, isolated total RNA, and performed qRT-PCR on four genes: *GPX2*, involved in detoxification, and *GSR*, *IDH1*, and *PGD*, involved in glutathione recycling. As expected, we observed significant upregulation of these genes between 30 and 60 mpi (Fig. 4d). Interestingly, Rai et al. showed that pneumococcal supernatant is sufficient to instigate DNA breaks caused by oxidative damage in A549 lung cells [50]. To test whether the glutathione-dependent reactive oxygen detoxification pathway could be upregulated by *S. pneumoniae* supernatant, we incubated epithelial cells with filtered pneumococcal supernatants. As shown in Fig. 4d, the genes were indeed activated. To establish that pneumococci-derived ROS was behind the response, we added the antioxidant resveratrol (100 μM) to the epithelial–pneumococcal model and did not observe activation (Fig. 4d). Together, these results partly explain the findings of Rai et al. [50] and reveal that lung epithelial cells try to counter the ROS produced by *S. pneumoniae* by upregulating glutathione biosynthesis.

### Pneumococcal transcriptional adaptation in response to epithelial cells

We clustered pneumococcal genes of the working library based on centered normalized gene expression in response to co-incubation with epithelial cells [35]. Clusters of genes were then categorized based on their reported functions [36, 50–52].

**Fig. 5:**
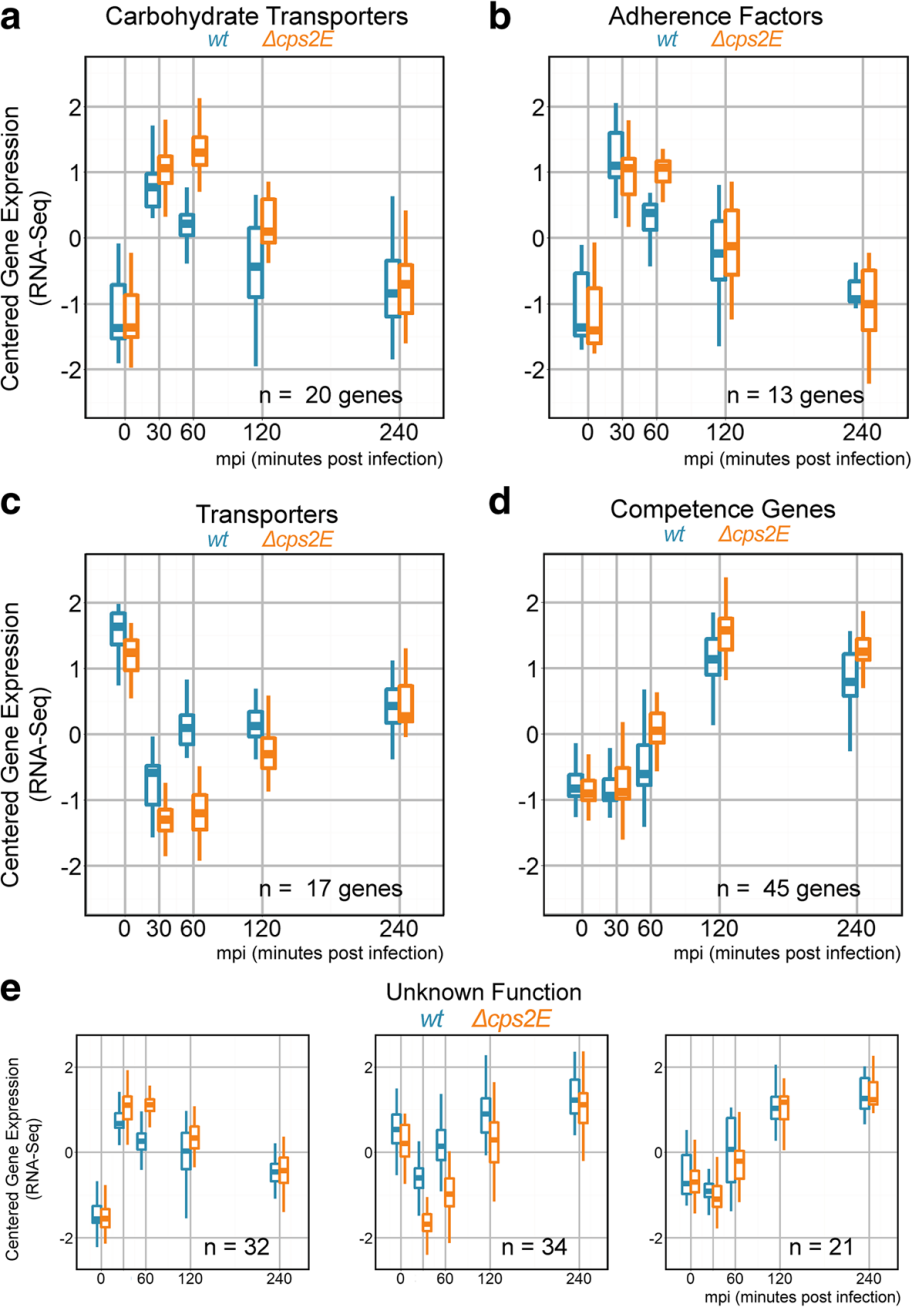
*S. pneumoniae* transcriptional adaptation in response to co-incubation with epithelial cells. We clustered pneumococcal genes based on centered normalized expression and recovered clusters of genes with shared function. **a** A subset of 20 genes encoding carbohydrate transporters displayed early activation at 30 mpi and returned to basal expression levels at later time points. The unencapsulated strain (*∆cps2E*, *orange box*) exhibited a more sustained expression up to 60 mpi than the wild type (*wt*). **b** Similarly, 13 genes encoding adherence factors showed the same profile of early activation at 30 mpi and a more sustained expression at 60 mpi in the unencapsulated strain. **c** Seventeen genes encoding non-carbohydrate transporters were repressed at 30 mpi. **d** And 45 genes that are part of the competence regulon were activated beginning at 60 mpi until the end of the experiment. **e** Interestingly, subsets of genes of unknown function showed mixed profiles, including early activation and repression followed by de-repression and late activation

One cluster displayed early gene activation at 30 mpi followed by a decrease in expression at later time points. This cluster contains 20 genes encoding carbohydrate transporters [52] (Fig. 5a) and 13 encoding adherence factors [53] (Fig. 5b). Six out of the 20 carbohydrate transporter genes were significantly activated (*p* < 0.05, DESeq2) at 60 mpi in the unencapsulated strain compared to the wild type (discussed below), highlighting a more sustained expression up to 60 mpi for the more adherent unencapsulated pneumococci. The adherence factors included *aliA*, *bgaA*, *amiA*, *rafE*, and *malX*, encoding well-known moonlighting proteins and pneumococcal virulence factors [54]. A previous array-based genome-wide transcriptional study has identified *aliA*, an oligopeptide transporter and adherence factor, as activated in an infection model involving another epithelial cell line, D562 [55]. *rafE*, another adherence factor and sugar-binding transporter, has also been reported to be part of pneumococcal genes activated early in response to epithelial cells [56].

Another gene cluster showed repression of gene expression at 30 mpi followed by increased expression at later time points. Seventeen genes encoding transporters are members of this cluster [36] (Fig. 5c). Various substrates have been reported to be transported by the transporters, including iron (SPD_0917/8), amino acids (*brnQ*), sugars (SPD_0740/1), and ions (SPD_1436). Song et al. [56] reported repression of SPD_0740, which encodes a sugar transporter, at the same time point (30 mpi). In our data, however, significant repression (*p* < 0.05) at 30 mpi occurred only in the unencapsulated and not the wild-type strain.

Figure 5d depicts a cluster of genes with a late activation profile starting at 60 mpi, continuing to 120 mpi and plateauing at 240 mpi. The profile is shared by 45 genes belonging to the competence regulon [51]. While Orihuela et al. [55] reported that only three competence genes (*ccs4*, *dprA*, and *cinA*) were activated in their study, we observed massive gene activation involving most of the competence genes in our model. Furthermore, we fused *gfp* to one of the competence genes, SPD_1711 (*ssbB*), under its own promoter and followed its expression using fluorescence microscopy. As shown in Fig. 3c, d, the protein level increased over time during early infection starting at 80 mpi, in line with increased gene expression occurring at 60 mpi.

Interestingly, a cluster containing genes of unknown function showed a varied profile, including early activation at 30 mpi, repression at 30 mpi, and later sustained activation starting at 60 mpi (Fig. 5e). Specifically, two genes (SPD_1426 and SPD_2043) were activated under our experimental conditions and were previously reported as being activated during infection using *S. pneumoniae* strain TIGR4 (SP1601 and SP2216, respectively) [56]. A complete list of genes and fold changes is available as Additional file 2: Table S1.

### Adherent *S. pneumoniae* repress the epithelial innate immune response

**Fig. 6:**
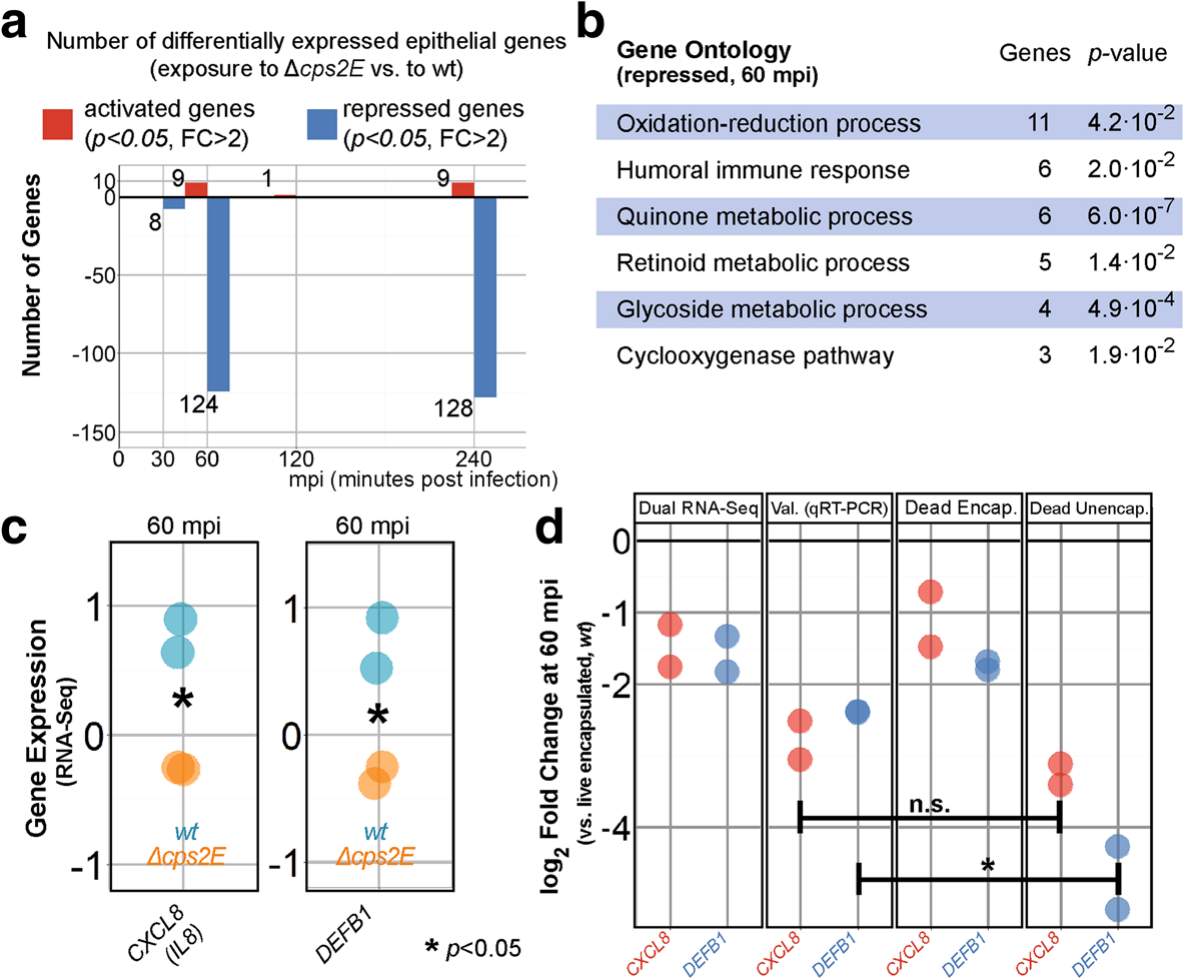
Adherent *S. pneumoniae* repress epithelial innate immune responses. **a** At 60 mpi, 124 epithelial genes were significantly repressed upon exposure to *∆cps2E* bacteria compared with wild-type pneumococci. **b** GO term enrichment analysis of 60-mpi repressed genes showed enrichment of “oxidation reduction” (11 genes, *p* = 4.2 × 10^–2^), “humoral immune response” (six genes, *p* = 2.0 × 10^–2^) and “quinone metabolic process” (six genes, *p* = 6.0 × 10^–7^) among others. **c** *∆cps2E-*exposed epithelial cells expressed 2.8 ± 1.3-fold less *CXCL8* and 3.0 ± 1.2-fold less *DEFB1* than epithelial cells exposed to wild type pneumococci. **d** We validated *CXCL8* and *DEFB1* repression by qRT-PCR. Heat-inactivated encapsulated bacteria showed no repression of *CXCL8* and *DEFB1*, i.e., no difference (*p* > 0.05) compared to viable encapsulated *S. pneumoniae* (*Dead Encap.*). While infection with heat-inactivated *∆cps2E* repressed *CXCL8* to the level of viable *∆cps2E*, *DEFB1* was more repressed (*p* < 0.05) by dead ∆*cps2E* than by viable unencapsulated pneumococci

Comparing epithelial gene expression in response to encapsulated and unencapsulated pneumococci allowed us to specifically identify adherence-responsive genes. In doing so, we identified 272 adherence-responsive (FC > 2, *p* < 0.05; DESeq2 analysis) epithelial genes, of which 19 were activated and 256 repressed during early infection (Fig. 6a). Three genes, *PTGS2*, *PTPRD*, and ENSG00000237831, showed repression and activation at more than one time point. GO term enrichment analysis of the subset of repressed genes at 60 mpi showed that the term “humoral immune response” was enriched, with six genes belonging to this category (*p* = 2.0 × 10^–2^; Fig. 6b).

*CXCL8* (*IL8*), encoding interleukin-8, was one of the repressed immunity genes. CXCL8 is a potent chemoattractant for neutrophil and other granulocytes. Interestingly, at 60 mpi, *∆cps2E*-exposed epithelial cells expressed 2.8 ± 1.2 less *CXCL8* than epithelial cells exposed to wild-type *S. pneumoniae* (Fig. 6c). This difference was validated by qRT-PCR (Fig. 6d). Further, we asked whether *CXCL8* repression is an active process or merely mediated by physical adherence. To assess this, we co-incubated heat-inactivated *∆cps2E* and heat-inactivated wild-type pneumococci with epithelial cells. Note that heat inactivation preserves pneumococcal epitope and protein structures [57]. *CXCL8* was still significantly repressed by dead *∆cps2E* but not by dead wild-type pneumococci (Fig. 6d), suggesting that *CXCL8* repression is independent of viability but dependent on the presence of the capsule or on the accessibility of surface-exposed (protein) factors in the absence of capsule. Intriguingly, Graham and Paton [58] showed that epithelial interleukin-8 production and release was suppressed by pneumococcal surface protein CbpA and incubation with *∆cbpA* leads to higher CXCL8 expression. We speculate that the absence of the capsule in *∆cps2E* increases accessibility of pneumococcal surface-exposed factors, including CbpA, to epithelial receptors, leading to repression of *CXCL8*.

*DEFB1*, encoding β-defensin-1, is an important epithelial-derived, constitutively expressed antimicrobial peptide [59]. In our model, however, *DEFB1* was not expressed constitutively but repressed 3.0 ± 1.2 times (*p* < 0.05, 60 mpi) in *∆cps2E*-exposed epithelial cells compared with wild type-exposed cells (Fig. 6c, validated in Fig. 6d). Additionally, while heat-inactivated wild type pneumococci stimulated comparable levels of *DEFB1* compared to viable wild-type bacteria, non-viable *∆cps2E* repressed *DEFB1* expression even more (*p* < 0.05) than viable *∆cps2E* (Fig. 6d). We conclude that *DEFB1* expression is affected by adherence, accessibility of pneumococcal surface proteins, and pneumococcal viability by an as of yet unknown mechanism. In summary, we show that adherent pneumococci modulate epithelial expression of innate immunity genes, including *CXCL8* and *DEFB1*, mediated by pneumococcal surface factors.

### Adherent *S. pneumoniae* activate sugar importers

**Fig. 7:**
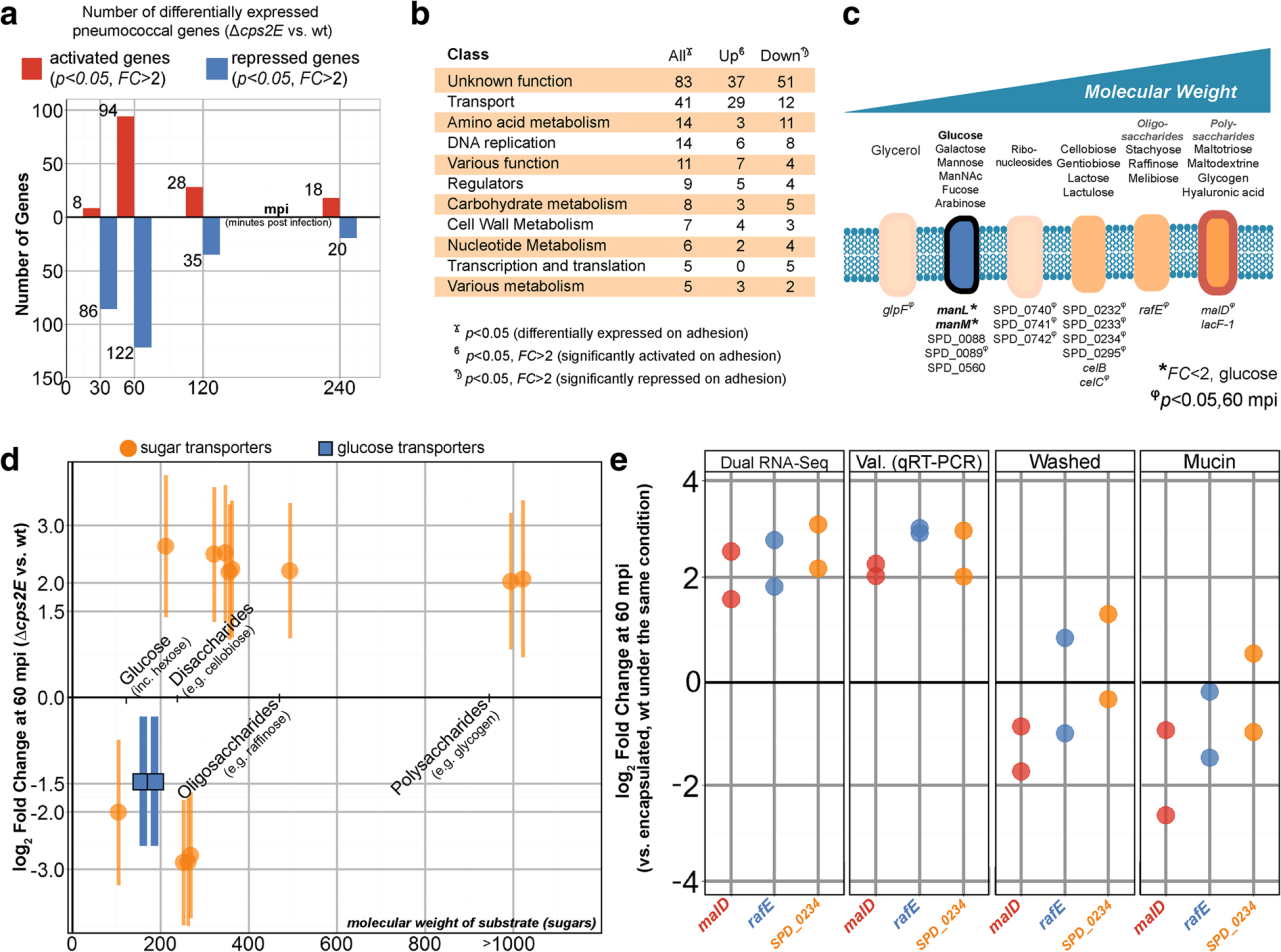
Adherent pneumococci gain access to host-derived carbohydrates and activate non-glucose sugar importers. **a** There were 295 genes differentially expressed between pneumococcal strains exposed to epithelial cells: 118 unique genes of the 295 genes were activated in *∆cps2E* compared to wild type (wt) pneumococci while 185 unique genes were repressed. Note that eight genes showed activation and repression at different time points. **b** Most of the differentially expressed genes are of unknown function (83 genes, 28 % of 295), followed by cellular transport (41 genes, 14 %), amino acid metabolism (14 genes, 5 %), and DNA replication, repair, and recombination (14 genes, 5 %). Note that an individual gene can be part of multiple classes. **c** Of the 41 transporter genes, 16 are described to transport carbohydrates. The carbohydrate importers transport a wide range of carbohydrates, from simple monosaccharides to complex polysaccharides. **d** At 60 mpi, the expression of glucose transporters (*manLM*, *blue boxes*) was repressed (*p* < 0.05, FC = 1.5) in *∆cps2E* compared to encapsulated *S. pneumoniae*. Eight non-glucose transporters were activated (*p* < 0.05, FC > 2) in the *∆cps2E* strain: SPD_0089, *celC*, SPD_0295, SPD_0232/33/34, *rafE*, and *malD*. **e** We validated the data by qRT-PCR for three sugar importers: *malD* (polysaccharides), *rafE* (oligosaccharide), and SPD_0234 (non-glucose disaccharide). By removing epithelial mucus prior to infection, the importers were no longer activated in *∆cps2E* compared to wild type (FC < 2, *Washed*). Incubation with type III porcine mucin (5 g/L) did not activate the genes in *∆cps2E* compared to encapsulated pneumococci (FC < 2)

Consistent with the epithelial cell analysis, we compared pneumococcal gene expression between unencapsulated and encapsulated libraries, resulting in 295 differentially expressed genes (*p* < 0.05). Specifically, of the 295 pneumococcal genes, 118 were activated (FC > 2) in the unencapsulated strain while 185 genes were repressed (FC > 2) (Fig. 7a). Six genes (SPD_0188, SPD_0194, SPD_1717, SPD_1751, SPD_1170, and SPD_1988) and two small non-coding RNAs (snRNAs; EBG0001438084 and EBG0001438080) showed repression and activation at different time points. Because the column-based RNA isolation used in our study does not efficiently extract sRNAs, we did not follow-up snRNAs in subsequent analysis (see “Methods”). We used gene classes developed for *S. pneumoniae* TIGR4 [37] to categorize adherence-responsive genes (Fig. 7b). Excitingly, most of the adherence-responsive genes are of unknown function (83 of 295 genes, 28 %), highlighting our paucity of knowledge on pneumococcal gene function. A large part of the subset (41 genes, 14 %) are involved in cellular transport (Fig. 7b). Since the pneumococcal genome has an exceptionally high number of carbohydrate transporters [52], it is not surprising that 16 of the genes encode sugar transporters (Fig. 7c).

At 60 mpi, 12 carbohydrate transporters were differentially expressed (*p* < 0.05, FC > 2) between *∆cps2E* and encapsulated pneumococci exposed to epithelial cells. In the presence of high glucose (2 g/L), *∆cps2E* expressed 1.5-fold less *manLM*, encoding glucose transporters, than encapsulated *S. pneumoniae* (Fig. 7d). Moreover, eight importers were activated in the adherent strain compared to the mostly free-floating encapsulated strain. The eight non-glucose transporter-genes and their substrates are SPD_0089 (disaccharides: galactose, mannose, N-acetylmannosamine), *celC* (disaccharides: cellobiose, gentiobiose), SPD_0232/33/34 (disaccharides: cellobiose), SPD_0295 (disaccharides: lactose and lactulose), *rafE* (oligosaccharides: raffinose, stachyose, melliobiose) and *malD* (polysaccharides: maltotitriol, maltodextrine, glycogen) (Fig. 7d). At the same time, four genes were repressed in unencapsulated *S. pneumoniae* exposed to human epithelial cells, *glpF* (glycerol) and SPD_0740/41/42 (ribonucleosides). We selected three transporter genes, *malD* (polysaccharides), *rafE* (oligosaccharides), and SPD_0234 (disaccharides), and validated the abovementioned observations by qRT-PCR (Fig. 7e).

Our data indicate that adherent unencapsulated bacteria detect non-glucose sugars in their immediate vicinity. Epithelial mucus may provide non-glucose carbohydrates [60, 61] and simultaneously limit the interaction of epithelial cells with encapsulated wild-type bacteria [62]. We then removed epithelial-associated mucus by washing the surface with warm PBS and observed that the genes were no longer activated (FC < 2; Fig. 7e). Next, we incubated pneumococcal strains with type III porcine mucin (5 g/L), mimicking complex carbohydrates in the medium. Interestingly, the importers were not differentially expressed between strains (FC < 2), indicating similar access to non-glucose sugars (Fig. 7e). We conclude that following adherence to epithelial cells, *S. pneumoniae* bacteria sense host-derived non-glucose carbohydrates and, in turn, activate expression of genes encoding transporters to import the now-available sugars.

## Discussion

Early infection is a complex and disruptive encounter between host and pathogen. In both species, a multitude of transcriptionally mediated cellular processes are fine-tuned, being activated, maintained, and repressed to ensure survival. The recently described dual RNA-seq approach allows simultaneous host–pathogen monitoring during their interaction [25–28]. In this study, we exploited the approach by applying dual RNA-seq to a model of pneumococcal infection of human lung alveolar epithelial cells. We have generated a detailed time-resolved dataset of epithelial–pneumococcal transcriptomes up to 4 h after infection. Moreover, we have validated the rich dataset by qRT-PCR and quantitative fluorescence microscopy. Furthermore, we have shown that adherence-specific transcriptional responses in host and pathogen can be identified by comparing the transcriptomes of lung epithelial cells in the presence of either encapsulated or unencapsulated pneumococci. Since adherence is the first step towards pathogenesis, adherence-specific regulated genes might provide interesting novel drug and/or vaccination targets that have not yet been picked up by conventional screens aimed at targeting essential and/or surface exposed proteins.

Our early infection model recapitulated three major in vivo characteristics of pneumococcal infection: pneumococcal adhesion, bacterial multiplication, and epithelial responses to the pneumococcus. Our model recapitulated these infection characteristics: (i) adherence for both encapsulated and unencapsulated pneumococci (Fig. 1); (ii) pneumococcal viability and growth during early infection, e.g., generation of ROS, activation of competence, and expression of carbohydrate importers (Figs. 4, 5 and 7); and (iii) host response to pneumococcal presence, e.g., glutathione-associated detoxification and innate immune responses (Figs. 4 and 6). Furthermore, as shown by upregulation of carbohydrate transporters (Fig. 7), pneumococci sense the presence of the epithelium and subsequently adapt their transcriptome.

Remarkably, we observed that all pneumococcal genes are expressed at one point or another during the infection process (Fig. 2). While this observation might partly stem from the high sequencing depth undertaken here (494 to 1588× for the pneumococcal genome, average coverage 1120×) the genome-wide bacterial gene expression confirms recent reports on bacteria adapting to multiple conditions [63, 64]. We speculate that interspecies interaction necessitates massive pneumococcal transcriptional adaptation. Moreover, we have observed activation of detoxification genes (*GPX2* and *GSR*) in epithelial cells, protecting these cells against pneumococci-derived ROS (Fig. 4). Indeed, *S. pneumoniae* has been reported to secrete high levels of peroxides as a by-product of its pyruvate metabolism [65] and has recently been shown to cause DNA damage-dependent apoptosis in alveolar lung epithelial cells [50]. Future work should also examine whether small non-coding RNAs play a role in pneumococcal early infection as they do in *Salmonella* [26], something beyond the scope of the current study. The hybrid column-based RNA isolation method we developed here to simultaneously recover high quality eukaryotic and prokaryotic total mRNA does not, unfortunately, allow the effective inclusion of small RNA species. In addition, the D39 genome is poorly annotated for snRNAs, further hindering differential sRNA analysis in our model. Future work may benefit from column-independent RNA isolation methods coupled with sufficient sequencing depth and transcriptome assembly to identify pneumococcal sRNAs important for infection.

Using array-based technology, Bootsma et al. [5] were the first to interrogate genome-wide transcriptional responses of human epithelial cells to *S. pneumoniae*. They reported that epithelial immunity and apoptosis genes are activated at 2 h post-infection. Here, we confirmed their observations and find that subsets of immunity and apoptotic genes displayed complex expression profiles in response to pneumococci, including repression followed by an immediate rebound, and activation followed by deactivation (Additional file 1: Figure S5 and Additional file 2: Table S6). In response to co-incubation with epithelial cells, pneumococcus adapts its transcription and adherence factors, transporters, and competence, among others, are activated (Fig. 5). Transcriptional activation of transporters has been reported in previous array-based studies of pneumococci infecting epithelial cells [6, 55, 56]. Nevertheless, by exploiting time-resolved simultaneous monitoring of host and pathogen, the dynamics underlying differential gene expression can be observed in greater detail. It should be noted that comparing specific results between two infection studies can be problematic. Aside from different technologies to monitor gene expression (qRT-PCR versus RNA-seq versus microarrays), numerous details, such as multiplicities of infection, history of epithelial cells, pre-treatment of the host and pathogen, infection medium, and pathogen strains can add layers to the complexity. In this regard, it is satisfying to note that many of the here-observed transcriptional responses have also been documented previously using different experimental set-ups.

### Outlook

The model used here could be expanded further by incorporating relevant LRTI agents. For example, alveolar macrophages and epithelial cells together form the epithelium lining the lower respiratory tract. The cells reciprocally influence cellular phenotypes and behaviors [66], highly relevant to infection. Moreover, pneumococcal co-infection and secondary infection with, for instance, influenza virus or *H. influenzae* are not unheard of [67–69]. Incorporation of other agents into the model and exploiting dual (or triple and quadruple) RNA-seq approaches may provide novel insights into respiratory infection.

Besides its relevance in communicable diseases, gained insights into pneumococcal infection are also applicable to understanding several non-communicable respiratory diseases. Asthma, the most common chronic respiratory disease, is a major risk factor for pneumococcal infection [70]. Additionally, chronic obstructive pulmonary disease (COPD) [71] and cigarette smoking [72] have been reported to increase the risk of pneumococcal LRTI. Here, we report the first study to show simultaneous transcriptomic changes of the pathogen *S. pneumoniae* and human lung alveolar epithelial cells during early infection, which might aid in identifying new biomarkers and drug targets to combat infection.

Though transcriptome rewiring is a focal point during interspecies interaction [22, 23], non-transcriptional regulation plays an important part during early infection. Capsule shedding, a hallmark of pneumococcal infection, is regulated by autolysin-A (LytA). LytA is activated when the bacterium encounters alveolar cathelicidin [17], which is independent of transcriptional regulation. Heterogeneity of cellular responses is another confounding factor [73]. Recently, dual RNA-seq combined with cell sorting was used to identify heterogeneous activity of *Salmonella* virulence factors that, in turn, drives a heterogeneous interferon response in macrophages [74]. This highlights the relevance of noise in gene expression and cell-to-cell variability in host–pathogen interactions. Furthermore, whole organism infection models offer a more systemic perspective. For instance, the dual RNA-seq approach has been used to monitor infection of wheat by bacteria [75] and mosquitoes by filaria [76]. Whole organism dual RNA-seq is not without its challenges, including averaging (host) effects to gene expression across all cell types.

## Conclusions

We have shown that a time-resolved dual RNA-seq approach can identify novel cellular processes during pneumococcal early infection. Furthermore, we have made the time-resolved dual transcriptomics dataset available to the broader research community (http://dualrnaseq.molgenrug.nl). We invite pneumococcal researchers to use the database to formulate research questions in the development of preventive and curative strategies against pneumococcal infection. Finally, we invite researchers from the fields of microbiology, immunology, and pulmonology to access the dataset and use it to develop their own hypotheses.

## Methods

### Culture of the epithelial cell line and *S. pneumoniae* D39 and pneumococcal transformation

Human type II lung epithelial cell line A549 (ATCC® CCL-185) and *S. pneumoniae* D39 were routinely cultured without antibiotics. Strain construction is described in detail in Additional file 1: Supplemental methods. Oligonucleotides are listed in Additional file 2: Table S2 and strains in Additional file 2: Table S3.

### Infection studies

Confluent monolayers of A549 were co-incubated with *S. pneumoniae* D39 at a MOI of 10 in 1 % fetal bovine serum in RPMI1640 medium without phenol red. Prior to infection, epithelial monolayers were kept for 10 more days after confluence. To optimize cell-to-cell contact, centrifugation was employed (2000 × g, 5 min, 4 °C). Adherence assays were performed by enumeration of plated colony-forming units in blood agar. More details are given in Additional file 1: Supplemental methods.

### Simultaneous total host–pathogen RNA isolation

On sterile, tissue culture-treated six-well plates (BD Falcon, The Netherlands), A549 was seeded until confluence and maintained (37 °C, 5 % (v/v) CO_2_) for another 10 days. Wild-type *S. pneumoniae* D39 and *∆cps2E* were grown in C + Y medium to OD_600nm_ ~0.2 and the medium was replaced with infection medium. As described, five time points were selected: 0, 30, 60, 120, and 240 mpi. Six technical replicates (individual wells) were pooled into one biological replicate. Two biological replicates were used for each time point, except for 240 mpi. To minimize transcriptional changes during sample handling, we did not separate the cellular mixture (epithelial cells, adherent pneumococci, and free-floating pneumococci). Rather, we simultaneously harvested the cells and isolated total RNA. To harvest the total RNA from host and pathogen at the same time, we treated the cellular mixture with a concentrated solution of ammonium sulfate to prevent any protein-dependent RNA degradation [77]. Each milliliter of the ammonium sulfate solution (pH 5.2) contained 0.7 g (NH_4_)_2_SO_4_. The saturated solution also contained 20 mM EDTA and 25 mM sodium citrate. Three parts of saturated solution of ammonium sulfate was added directly to one part of medium. The suspension was vigorously pipetted to ensure the complete mixing of the ammonium sulfate solution and infection medium. Adherent host cells were scraped off (sterile, TPP, VWR, The Netherlands) and incubated further (room temperature, 5 min). The suspension was collected and centrifuged at full speed (20 min, 4 °C, 10,000 × g). The supernatant was removed and the cell pellet was snap-frozen with liquid nitrogen. The cell mixture contained host cells, adherent bacteria, and non-adherent bacteria; the last two fractions were of varying proportions depending on the duration of co-incubation and the absence/presence of capsule.

To disrupt cells, bead beating was used. In a 1.5 ml screw cap tube, a PCR tube full of sterile, RNase-free glass beads (100 μm) were added together with 50 μl 10 % SDS and 500 μl phenol-chloroform. In the meantime, the frozen cell pellet was resuspended in TE solution (10 mM Tris-HCl, 1 mM Na_2_DTA, pH 8.0) treated with DEPC (diethylpyrocarbonate). The cell suspension was added into the screw cap tube and bead beaten three times for 45 s each. Tubes were immediately placed on ice and centrifuged at full speed at 4 °C to separate the organic and aqueous phases. The aqueous phase was pipetted out and back-extraction was performed on the organic phase to optimize RNA yield. The subsequent part of the RNA isolation was performed based on the High Pure RNA Isolation Kit (Roche, The Netherlands). The aqueous phase from the phenol-chloroform extraction was mixed well with binding buffer and pipetted into the upper chamber of the column and centrifuged. DNase mix was then added onto the filter and incubated at room temperature for 30 min to digest total genomic DNA. Total RNA was eluted according to the manufacturer’s protocol. The quality of total RNA was assayed by Nanodrop and a 1 % bleach gel was employed to interrogate genomic DNA and host–pathogen rRNA bands (host, 28S, 5 kbp and 18S, 1.8 kbp; pathogen, 23S, 2.9 kbp and 16S, 1.5 kbp). Relative enrichment of pneumococcal reads in dual RNA-seq may stem from the total RNA isolation protocol. We developed the protocol from an existing protocol for pneumococcal RNA isolation. We expanded this protocol to accommodate RNA isolation from epithelial cells. We speculate that the first step involving organic–aqueous liquid–liquid separation may favor isolating more pneumococcal RNAs with different characteristics than epithelial RNAs. RNA liquid–liquid separation ultimately depended on pH and ion strength of the aqueous phase [78].

### Library preparation, sequencing, data pipeline, and online database

Human and pneumococcal ribosomal RNAs were simultaneously depleted (“dual rRNA depletion”) by a 1:1 mixture of human/mouse/rat and Gram-positive bacteria capture probes (Ribo-Zero rRNA Removal Kits, Illumina, US). Stranded cDNA library preparation was performed with the TruSeq® Stranded Total RNA Sample Preparation Kit (Illumina, US) according to the manufacturer’s protocol. cDNA sequencing of the 18 samples was performed in three lanes (six samples per lane) of Illumina NextSeq 500 with a HighOutput Flowcell in 75 single end mode. Samples were de-multiplexed and analyzed further. The raw fastq data are accessible at http://www.ncbi.nlm.nih.gov/geo/ with accession number GSE79595.

The quality of raw reads was checked by FastQC v0.11.5 (Babraham Bioinformatics, UK) [79]. To improve the quality of reads and remove adapter sequences, we trimmed the reads in single end mode using these criteria: (i) adapter sequences removed based on TruSeq3-SE library; (ii) leading nucleotide with low quality removed; (iii) low quality trailing nucleotide removed; (iv) a five-nucleotide sliding window in which the average quality score must be above 20; and (v) with a minimum read length of 50 [31]. The trimmed reads were again checked by FastQC. To align the reads, we generated a chimeric genome by concatenating the *S. pneumoniae* D39 genome (ENSEMBL, release 31, bacteria 13 collection, date of download 13 June 2016) [33] as an extra chromosome of *Homo sapiens* (ENSEMBL, release 84, date of download 13 June 2016) [33]. The corresponding annotation files were downloaded from the aforementioned repositories. To ensure an undisrupted mapping of SPD_0001, a pneumococcal gene located at the very beginning of the pneumococcal genome sequence file, we copied the first 600 bp of the pneumococcal genome to the end of the genome and annotated this extra copy as SPD*_*0001a. Alignment of trimmed reads to the chimeric genome was performed by STAR, with the following options: (i) alignIntronMax 1 and (ii) sjdbOverhang 49 [32]. The subsequently mapped reads were then counted [34] according to the chimeric annotation file in (i) multimapping mode (-M), in which fractional count was reported (--fraction), (ii) allowing for overlapping reads across features to accommodate bacterial operons (iii) in a stranded option.

Subsequently, we separately analyzed host and pathogen libraries in R-studio (R v3.3.1). We performed differential gene expression analysis on rounded raw count by DESeq2 [41]. Epithelial and pneumococcal libraries were normalized by DESeq2: epithelial reads by regularized logarithm and pneumococcal reads by variance-stabilizing transformation. Aside from DESeq2-transformed counts, the rounded raw counts were transformed into TPM (transcripts per million) and log-transformed TPM [42]. The three transformed counts are used to visualize gene expression level in our online database (http://dualrnaseq.molgenrug.nl).

Unexpressed genes were removed from the working libraries, i.e., genes with no reads in any of the libraries. Furthermore, we removed genes with significant differences between unencapsulated (*∆cps2E*) and encapsulated (*wt*) libraries at 0 mpi and genes with no significance (*p* > 0.05) and FC < 2 from the aforementioned contrasts (Additional file 1: Figure S3). Genes with significant fold changes (*p* > 0.05, FC > 2) are listed in Additional file 2: Table S4: Epithelial differential gene expression and Additional file 2: Table S5: Pneumococcal differential gene expression. NA notes FC < 2.

Soft clustering was used on normalized centered gene expression values with a fuzzifier value of 1.5 to obtain a better view of the dynamics of gene expression during infection [35]. The number of clusters was approximated by a built-in function and further adjusted until a suitable number of clusters appeared, i.e., without any empty group. Enrichment of GO for host genes was analyzed by amiGO ver. 2.4 [36].

The database can be accessed at http://dualrnaseq.molgenrug.nl. The data are stored in a MySQL database containing both human and *S. pneumoniae* gene expression values. Gene expression graphs during early infection are generated by D3 (Data Driven Documents, https://d3js.org). Gene expression is presented in DESeq2-normalized values [41], TPM (transcripts per million) [42] or log-transformed TPM.

### qRT-PCR and quantitative fluorescence microscopy

Infection studies were repeated, total RNA isolated, and qRT-PCR performed. For fluorescence microscopy, infection studies were performed in eight-well μ-slides (Ibidi, Germany). More details are provided in Additional file 1: Supplemental methods.

## Additional files

Additional file 1: Supplemental information. All supplemental figures, supplemental methods and Tables S2. and S3. (DOCX 1.19 mb)
Additional file 2: Supplemental tables. Tables S1, S4, S5 and S6. (XLSX 607 kb)

## References

[1] Prina E, Ranzani OT, Torres A (2015). Community-acquired pneumonia. Lancet.

[2] Kadioglu A, Weiser JN, Paton JC, Andrew PW (2008). The role of *Streptococcus pneumoniae* virulence factors in host respiratory colonization and disease. Nat Rev Microbiol.

[3] Hammerschmidt S, Bergmann S, Paterson GK, Mitchell TJ (2007). Pathogenesis of *Streptococcus pneumoniae* infections: adaptive immunity, innate immunity, cell biology, virulence factors. Community-acquired pneumonia, Birkhäuser advances in infectious diseases.

[4] Lee H-Y, Andalibi A, Webster P, Moon S-K, Teufert K, Kang S-H (2004). Antimicrobial activity of innate immune molecules against *Streptococcus pneumoniae*, *Moraxella catarrhalis* and nontypeable *Haemophilus influenzae*. BMC Infect Dis.

[5] Bootsma HJ, Egmont-Petersen M, Hermans PWM (2007). Analysis of the in vitro transcriptional response of human pharyngeal epithelial cells to adherent *Streptococcus pneumoniae*: evidence for a distinct response to encapsulated strains. Infect Immun.

[6] Mlacha SZK, Romero-Steiner S, Hotopp JCD, Kumar N, Ishmael N, Riley DR (2013). Phenotypic, genomic, and transcriptional characterization of *Streptococcus pneumoniae* interacting with human pharyngeal cells. BMC Genomics.

[7] Westermann AJ, Gorski SA, Vogel J (2012). Dual RNA-seq of pathogen and host. Nat Rev Microbiol.

[8] Voynow JA, Rubin BK (2009). Mucins, mucus, and sputum. Chest.

[9] Rose MC, Voynow JA (2006). Respiratory tract mucin genes and mucin glycoproteins in health and disease. Physiol Rev.

[10] Tecle T, Tripathi S, Hartshorn KL (2010). Defensins and cathelicidins in lung immunity. Innate Immun.

[11] Hallstrand TS, Hackett TL, Altemeier WA, Matute-Bello G, Hansbro PM, Knight DA (2014). Airway epithelial regulation of pulmonary immune homeostasis and inflammation. Clin Immunol.

[12] Soumelis V, Reche PA, Kanzler H, Yuan W, Edward G, Homey B (2002). Human epithelial cells trigger dendritic cell mediated allergic inflammation by producing TSLP. Nat Immunol.

[13] Bogaert D, De Groot R, Hermans PWM (2004). *Streptococcus pneumoniae* colonisation: the key to pneumococcal disease. Lancet Infect Dis.

[14] Abeyta M, Hardy GG, Yother J (2003). Genetic alteration of capsule type but not PspA type affects accessibility of surface-bound complement and surface antigens of *Streptococcus pneumoniae*. Infect Immun.

[15] Hyams C, Camberlein E, Cohen JM, Bax K, Brown JS (2010). The *Streptococcus pneumoniae* capsule inhibits complement activity and neutrophil phagocytosis by multiple mechanisms. Infect Immun.

[16] Beiter K, Wartha F, Hurwitz R, Normark S, Zychlinsky A, Henriques-Normark B (2008). The Capsule sensitizes *Streptococcus pneumoniae* to α-defensins human neutrophil proteins 1 to 3. Infect Immun.

[17] Kietzman CC, Gao G, Mann B, Myers L, Tuomanen EI (2016). Dynamic capsule restructuring by the main pneumococcal autolysin LytA in response to the epithelium. Nat Commun..

[18] Shelburne SA, Davenport MT, Keith DB, Musser JM (2008). The role of complex carbohydrate catabolism in the pathogenesis of invasive streptococci. Trends Microbiol.

[19] Rajam G, Anderton JM, Carlone GM, Sampson JS, Ades EW (2008). Pneumococcal surface adhesin A (PsaA): a review. Crit Rev Microbiol.

[20] Gray RD, Duncan A, Noble D, Imrie M, O’Reilly DSJ, Innes JA (2010). Sputum trace metals are biomarkers of inflammatory and suppurative lung disease. Chest.

[21] Tseng H-J, McEwan AG, Paton JC, Jennings MP (2002). Virulence of *Streptococcus pneumoniae*: PsaA mutants are hypersensitive to oxidative stress. Infect Immun.

[22] Jenner RG, Young RA (2005). Insights into host responses against pathogens from transcriptional profiling. Nat Rev Microbiol.

[23] Sorek R, Cossart P (2010). Prokaryotic transcriptomics: a new view on regulation, physiology and pathogenicity. Nat Rev Genet.

[24] Kukurba KR, Montgomery SB. RNA sequencing and analysis. Cold Spring Harb Protoc. 2015;2015(11):pdb.top084970. 10.1101/pdb.top084970 PMC4863231 25870306

[25] Tierney L, Linde J, Müller S, Brunke S, Molina JC, Hube B (2012). An interspecies regulatory network inferred from simultaneous RNA-seq of Candida albicans invading innate immune cells. Front Microbiol..

[26] Westermann AJ, Förstner KU, Amman F, Barquist L, Chao Y, Schulte LN (2016). Dual RNA-seq unveils noncoding RNA functions in host-pathogen interactions. Nature.

[27] Baddal B, Muzzi A, Censini S, Calogero RA, Torricelli G (2015). Guidotti S, et al. Dual RNA-seq of nontypeable Haemophilus influenzae and host cell transcriptomes reveals novel insights into host-pathogen cross talk. mBio..

[28] Dillon LAL, Suresh R, Okrah K, Corrada Bravo H, Mosser DM, El-Sayed NM (2015). Simultaneous transcriptional profiling of *Leishmania major* and its murine macrophage host cell reveals insights into host-pathogen interactions. BMC Genomics..

[29] Pedersen M, Nissen S, Mitarai N, Svenningsen SL, Sneppen K, Pedersen S (2011). The functional half-life of an mRNA depends on the ribosome spacing in an early coding region. J Mol Biol.

[30] Kjos M, Aprianto R, Fernandes VE, Andrew PW, van Strijp JAG, Nijland R (2015). Bright fluorescent *Streptococcus pneumoniae* for live-cell imaging of host-pathogen interactions. J Bacteriol.

[31] Bolger AM, Lohse M, Usadel B. Trimmomatic: A flexible trimmer for Illumina sequence data. Bioinformatics. 2014;30(15):2114–20. 10.1093/bioinformatics/btu170 PMC4103590 24695404

[32] Dobin A, Davis CA, Schlesinger F, Drenkow J, Zaleski C, Jha S (2013). STAR: ultrafast universal RNA-seq aligner. Bioinformatics.

[33] Yates A, Akanni W, Amode MR, Barrell D, Billis K, Carvalho-Silva D (2016). Ensembl 2016. Nucleic Acids Res.

[34] Liao Y, Smyth GK, Shi W (2014). featureCounts: an efficient general purpose program for assigning sequence reads to genomic features. Bioinformatics.

[35] Kumar L, E. Futschik M (2007). Mfuzz: A software package for soft clustering of microarray data. Bioinformation.

[36] Carbon S, Ireland A, Mungall CJ, Shu S, Marshall B, Lewis S (2009). AmiGO: online access to ontology and annotation data. Bioinformatics.

[37] van Opijnen T, Camilli A (2012). A fine scale phenotype-genotype virulence map of a bacterial pathogen. Genome Res.

[38] The ENCODE Consortium. Standards, guidelines and best practices for RNA-seq: 2010/2011. http://bit.ly/29l4ihE. Accessed 10 June 2016.

[39] Hackett NR, Butler MW, Shaykhiev R, Salit J, Omberg L, Rodriguez-Flores JL (2012). RNA-seq quantification of the human small airway epithelium transcriptome. BMC Genomics..

[40] St-Pierre C, Brochu S, Vanegas JR, Dumont-Lagacé M, Lemieux S, Perreault C (2013). Transcriptome sequencing of neonatal thymic epithelial cells. Sci Rep..

[41] Love MI, Huber W, Anders S (2014). Moderated estimation of fold change and dispersion for RNA-seq data with DESeq2. Genome Biol..

[42] Wagner GP, Kin K, Lynch VJ (2012). Measurement of mRNA abundance using RNA-seq data: RPKM measure is inconsistent among samples. Theory Biosci Theor Den Biowissenschaften.

[43] Livak KJ, Schmittgen TD (2001). Analysis of relative gene expression data using real-time quantitative PCR and the 2(-Delta Delta C(T)) method. Methods.

[44] Ning K, Fermin D, Nesvizhskii AI (2012). Comparative analysis of different label-free mass spectrometry based protein abundance estimates and their correlation with RNA-seq gene expression data. J Proteome Res.

[45] Taniguchi Y, Choi PJ, Li G-W, Chen H, Babu M, Hearn J (2010). Quantifying *E. coli* proteome and transcriptome with single-molecule sensitivity in single cells. Science.

[46] Attaiech L, Olivier A, Mortier-Barrière I, Soulet A-L, Granadel C, Martin B (2011). Role of the single-stranded DNA-binding protein SsbB in pneumococcal transformation: maintenance of a reservoir for genetic plasticity. PLoS Genet.

[47] Vogel C, Marcotte EM (2012). Insights into the regulation of protein abundance from proteomic and transcriptomic analyses. Nat Rev Genet.

[48] Valko M, Leibfritz D, Moncol J, Cronin MTD, Mazur M, Telser J (2007). Free radicals and antioxidants in normal physiological functions and human disease. Int J Biochem Cell Biol.

[49] Forman HJ, Zhang H, Rinna A (2009). Glutathione: overview of its protective roles, measurement, and biosynthesis. Mol Aspects Med.

[50] Rai P, Parrish M, Tay IJJ, Li N, Ackerman S, He F (2015). *Streptococcus pneumoniae* secretes hydrogen peroxide leading to DNA damage and apoptosis in lung cells. Proc Natl Acad Sci U S A.

[51] Martin B, Prudhomme M, Alloing G, Granadel C, Claverys JP (2000). Cross-regulation of competence pheromone production and export in the early control of transformation in *Streptococcus pneumoniae*. Mol Microbiol.

[52] Bidossi A, Mulas L, Decorosi F, Colomba L, Ricci S, Pozzi G (2012). A functional genomics approach to establish the complement of carbohydrate transporters in *Streptococcus pneumoniae*. PLoS One.

[53] Pérez-Dorado I, Galan-Bartual S, Hermoso JA (2012). Pneumococcal surface proteins: when the whole is greater than the sum of its parts. Mol Oral Microbiol.

[54] Henderson B, Martin A (2011). Bacterial virulence in the moonlight: multitasking bacterial moonlighting proteins are virulence determinants in infectious disease. Infect Immun.

[55] Orihuela CJ, Radin JN, Sublett JE, Gao G, Kaushal D, Tuomanen EI (2004). Microarray analysis of pneumococcal gene expression during invasive disease. Infect Immun.

[56] Song X-M, Connor W, Hokamp K, Babiuk LA, Potter AA (2008). *Streptococcus pneumoniae* early response genes to human lung epithelial cells. BMC Res Notes.

[57] Hvalbye BKR, Aaberge IS, Løvik M, Haneberg B (1999). Intranasal immunization with heat-inactivated *Streptococcus pneumoniae* protects mice against systemic pneumococcal infection. Infect Immun.

[58] Graham RMA, Paton JC (2006). Differential role of CbpA and PspA in modulation of in vitro CXC chemokine responses of respiratory epithelial cells to infection with *Streptococcus pneumoniae*. Infect Immun.

[59] Krisanaprakornkit S, Weinberg A, Perez CN, Dale BA (1998). Expression of the peptide antibiotic human beta-defensin 1 in cultured gingival epithelial cells and gingival tissue. Infect Immun.

[60] Yesilkaya H, Manco S, Kadioglu A, Terra VS, Andrew PW (2008). The ability to utilize mucin affects the regulation of virulence gene expression in *Streptococcus pneumoniae*. FEMS Microbiol Lett.

[61] Paixão L, Oliveira J, Veríssimo A, Vinga S, Lourenço EC, Ventura MR (2015). Host glycan sugar-specific pathways in *Streptococcus pneumoniae*: galactose as a key sugar in colonisation and infection [corrected]. PLoS One.

[62] Nelson AL, Roche AM, Gould JM, Chim K, Ratner AJ, Weiser JN (2007). Capsule enhances pneumococcal colonization by limiting mucus-mediated clearance. Infect Immun.

[63] Kröger C, Colgan A, Srikumar S, Händler K, Sivasankaran SK, Hammarlöf DL (2013). An Infection-Relevant Transcriptomic Compendium for *Salmonella enterica* Serovar Typhimurium. Cell Host Microbe.

[64] Nicolas P, Mäder U, Dervyn E, Rochat T, Leduc A, Pigeonneau N (2012). Condition-dependent transcriptome reveals high-level regulatory architecture in *Bacillus subtilis*. Science.

[65] Carvalho SM, Kloosterman TG, Kuipers OP, Neves AR (2011). CcpA ensures optimal metabolic fitness of *Streptococcus pneumoniae*. PLoS One.

[66] Hussell T, Bell TJ (2014). Alveolar macrophages: plasticity in a tissue-specific context. Nat Rev Immunol.

[67] Siegel SJ, Roche AM, Weiser JN (2014). Influenza promotes pneumococcal growth during coinfection by providing host sialylated substrates as a nutrient source. Cell Host Microbe.

[68] Kash JC, Walters K-A, Davis AS, Sandouk A, Schwartzman LM, Jagger BW, et al. Lethal synergism of 2009 pandemic H1N1 influenza virus and *Streptococcus pneumoniae* coinfection is associated with loss of murine lung repair responses. mBio. 2011;2(5):e00172–11. 10.1128/mBio.00172-11 PMC3175626 21933918

[69] Lysenko ES, Lijek RS, Brown SP, Weiser JN (2010). Within-host competition drives selection for the capsule virulence determinant of *Streptococcus pneumoniae*. Curr Biol.

[70] Talbot TR, Hartert TV, Mitchel E, Halasa NB, Arbogast PG, Poehling KA (2005). Asthma as a risk factor for invasive pneumococcal disease. N Engl J Med.

[71] Decramer M, Janssens W, Miravitlles M (2012). Chronic obstructive pulmonary disease. Lancet.

[72] Phipps JC, Aronoff DM, Curtis JL, Goel D, O’Brien E, Mancuso P (2010). Cigarette smoke exposure impairs pulmonary bacterial clearance and alveolar macrophage complement-mediated phagocytosis of *Streptococcus pneumoniae*. Infect Immun.

[73] Jørgensen MG, van Raaphorst R, Veening J-W. Noise and stochasticity in gene expression: a pathogenic fate determinant. In: Harwood C and Wipat A, editor. Methods in microbiology. Oxford: Academic Press; 2013. p. 157–75. (Microbial Synthetic Biology; vol. 40).

[74] Avraham R, Haseley N, Brown D, Penaranda C, Jijon HB, Trombetta JJ (2015). Pathogen cell-to-cell variability drives heterogeneity in host immune responses. Cell.

[75] Camilios-Neto D, Bonato P, Wassem R, Tadra-Sfeir MZ, Brusamarello-Santos LC, Valdameri G (2014). Dual RNA-seq transcriptional analysis of wheat roots colonized by *Azospirillum brasilense* reveals up-regulation of nutrient acquisition and cell cycle genes. BMC Genomics..

[76] Choi Y-J, Aliota MT, Mayhew GF, Erickson SM, Christensen BM (2014). Dual RNA-seq of parasite and host reveals gene expression dynamics during filarial worm–mosquito interactions. PLoS Negl Trop Dis.

[77] Korfhage C, Wyrich R, Oelmuller U. Ammonium sulfate for neutralization of inhibitory effects. Google Patents; 2002. http://bit.ly/29qwG3E. Accessed 10 June 2016.

[78] Chomczynski P, Sacchi N (2006). The single-step method of RNA isolation by acid guanidinium thiocyanate–phenol–chloroform extraction: twenty-something years on. Nat Protoc.

[79] Andrews S. FastQC: a quality control tool for high throughput sequence data. 2010. http://bit.ly/29caLYC. Accessed 10 June 2016.

