# Supplementary material for: Time-resolved dual RNA-seq reveals extensive rewiring of lung epithelial and pneumococcal transcriptomes during early infection
Source: Genome Biol. 2016 Sep 27;17:198. doi: 10.1186/s13059-016-1054-5 (PMC5039909; doi:10.1186/s13059-016-1054-5)
Supplement: Additional file 1: — Supplemental information. All supplemental figures, supplemental methods and Tables S2. and S3. (DOCX 1.19 mb) [file 13059_2016_1054_MOESM1_ESM.docx]

**Supplemental information to:**

**Time-resolved Dual RNA-Seq Reveals Extensive Rewiring of Lung Epithelial and Pneumococcal Transcriptomes during Early Infection**

Rieza Aprianto^1^, Jelle Slager^1^, Siger Holsappel^1^ and Jan-Willem Veening^1,^*

^1^Molecular Genetics Group, Groningen Biomolecular Sciences and Biotechnology Institute, Centre for Synthetic Biology, University of Groningen, Nijenborgh 7, 9747 AG Groningen, The Netherlands.

^2^Department of Fundamental Microbiology, Faculty of Biology and Medicine,

University of Lausanne, Biophore Building, CH-1015 Lausanne, Switzerland.

^*^Correspondence to: Jan-Willem.Veening@unil.ch

**
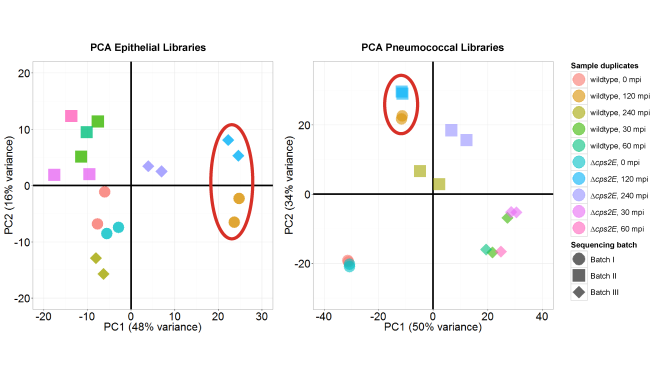
**

| **Fig S1.** Principal Component Analysis was performed separately to epithelial and pneumococcal libraries. Biological duplicates clustered closely to each other in host and pathogen libraries (see sample duplicates). Variability within samples were mainly dependent of the infection time points but not sequencing batch of the samples (see samples inside red circles). |
| --- |
|  |


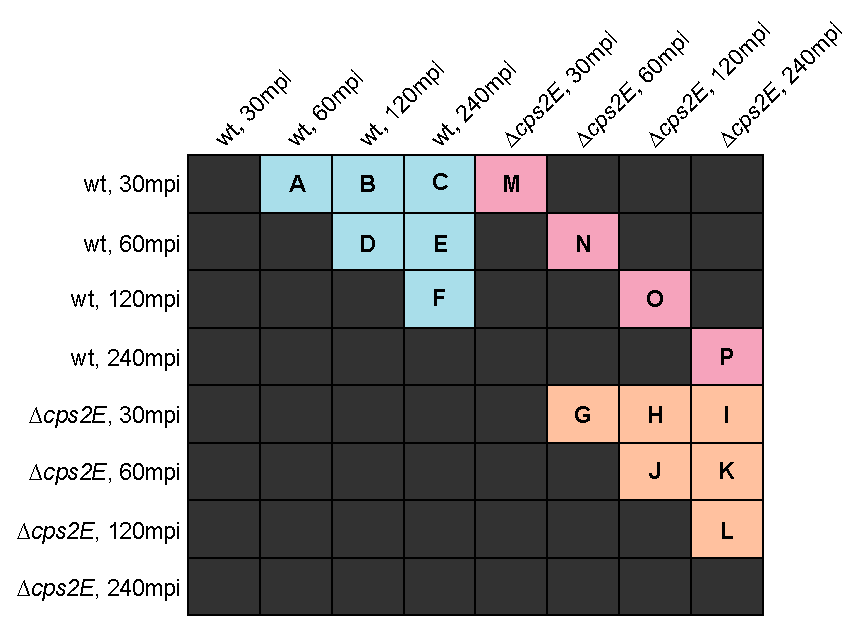


| **Fig S2.**  DESeq2 contrasts to compare differentially expressed genes in early infection |  |
| --- | --- |
| **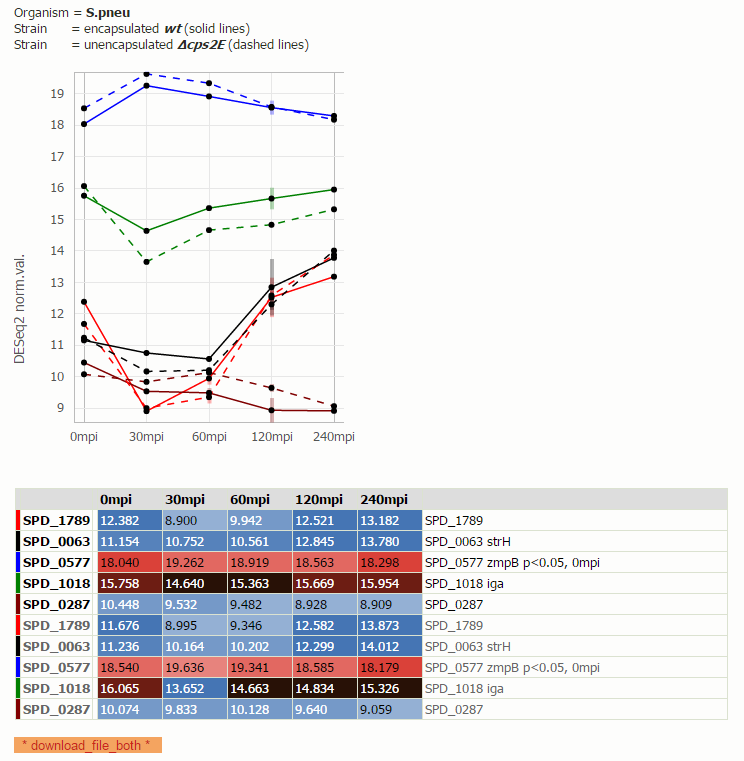** | |
| **Fig S3. Screenshots of the online dual RNA-Seq database at** <http://dualrnaseq.molgenrug.nl>.  Examples of the online database. Multiple genes of interest can be queried at the same time. Pneumococcal LPxTG surface proteins visualized with DESeq2 normalized value.  For visualization of gene expression during early infection, users can choose between DESeq2 normalization (1), TPM (transcript-per-million (2) or log-transformed TPM values. Users can adjust the size of the graph (in pixels) and the color scheme for the generated heatmap. Simply, enter multiple gene(s) of interest in the search bar and press “add” to query.  Furthermore, users can hover over the graph to see the actual values of the points and click the NCBI link for more information on the particular gene. Users can download the data file by pressing “download”. Three graphs and tables are generated per query, i.e., for expression in encapsulated (wt) libraries and unencapsulated (*∆cps2E*) libraries individually and for expression in combined libraries. | |

| **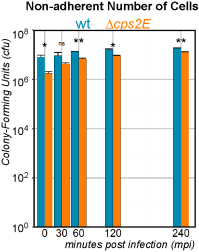** |
| --- |
| **Fig. S4. Number of non-adherent pneumococcal cells through co-incubation.** Number of free-floating cells, regardless of strains, increases over time in the medium showing pneumococci capability to multiply in the model, recapitulating a hallmark of infection. |
| **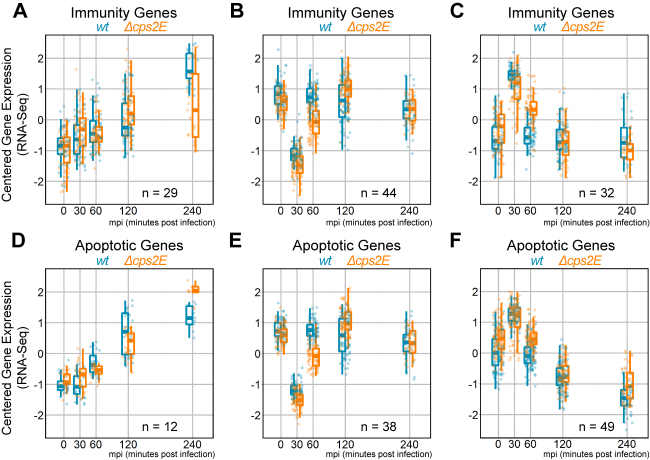** |
| **Fig. S5. Expression of epithelial immunity and apoptotic genes in response to co-incubation with *S. pneumoniae* strains.** We extracted immunity and apoptotic genes from epithelial working library and clustered the genes based on its centered normalized expression during early infection (3). Gene expression values are presented both as boxplot and scattered plot grouped by exposure to unencapsulated and wildtype pneumococci and time points. Gene list along with fold change are available in **Additional file 2: Table S6.** Details of boxplot: lowest whisker (smallest observation ≥ lower hinge – 1.5 · IQR (inter-quartile range); lower hinge (first quartile, quartile 25%); middle (median, 50% quartile); upper hinge (third quartile, quartile 75%) and highest whisker (largest observation ≤ upper hinge + 1.5 · IQR).  **A.** Immunity genes with activated trend throughout early infection. **B.** Immunity genes repressed at 30 mpi, returns to higher expression at 60 mpi for exposure to encapsulated wildtype pneumococci and at 120 mpi on epithelial cells exposed to unencapsulated pneumococci. **C**. Immunity genes with activation 30 mpi upon exposure to pneumococci. Gene expression returns at 60 mpi for epithelial cells exposed to encapsulated wildtype bacteria and at 120 mpi for epithelial cells exposed to unencapsulated more-adherent pneumococci. **D**. Apoptotic genes with activation trends throughout co-incubation. **E**. Apoptotic genes with repression at 30 mpi. Gene expression returns after 60 mpi for epithelial cells exposed to wildtype pneumococci and 120 mpi for exposed to unencapsulated bacteria. **F**. Activated apoptotic genes at 30 mpi for exposure to both pneumococcal strains. |

**Supplemental Methods**

**Culturing of human epithelial cell line, A549**

The human type II lung epithelial cell line, A549 (ATCC® CCL-185) was routinely cultured in DMEM (Dulbecco’s modified Eagle medium–nutrient mixture) F-12 with GlutaMAX (Life Technologies, NL) supplemented with 10% (v/v) fetal bovine serum (FBS; VWR, NL). The cell culture was maintained at humidified 5% (v/v) CO_2_ atmosphere at 37°C on sterile, tissue-culture treated plastic flasks (BD Falcon, VWR, NL) with a seeding dilution of 1:25. For all experiments, we re-seeded the A549 into sterile, tissue-culture treated plastic containers (BD Falcon, VWR, NL) at confluent density of 1.5 · 10^6^ cells·cm^-2^. After confirming confluence by light microscopy, the culture was kept for another 10 days to allow epithelial polarization. Passage and seeding of epithelial cell culture were performed without antibiotics to avoid any antibiotics residue during infection studies (5).

**Culture of *Streptococcus* *pneumoniae* and transformation**

*S. pneumoniae* strains were grown from frozen culture in liquid casein-based medium (pH 6.8), supplemented with yeast extract (Sigma-Aldrich, Boom, NL) (C+Y) at 37°C. For infection studies, cultures were grown until OD_600_ ~0.2 (exponential phase). For transformation, cells were grown until OD_600_ ~0.1. Subsequently, synthetic CSP-1 (100 ng·ml^-1^, competence-stimulating peptide 1) was added into the suspension and incubation was continued for another 12 min at 37°C.

Transforming DNA was added to the now-competent *S. pneumoniae*. Cells were then incubated at 30°C for 20 min to allow for internalization of DNA. Afterwards, fresh C+Y was added and the suspension was incubated further at 37°C for 90 minutes. Selection of transformants was done by plating in Columbia agar (Oxoid, UK) supplemented 2% with sheep blood (Johnny Rottier, NL) and 100 μg·ml^-1^ spectinomycin. The following day, colonies were streaked on spectinomycin-supplemented blood agar and incubated in 5% (v/v) CO_2_, overnight. Constructs were checked by colony PCR, Sanger sequencing and/or inspection of GFP expression by fluorescence microscopy. Growth curves for mutants were monitored with a Tecan Infinite 200 Pro Microtiter Plate Reader in 96-wells plates (5).

**Generation of pneumococcal *gfp*-fusion strains**

Four genes, i.e. *SPD_0475*, *SPD_0963*, *SPD*_*1711* and *SPD_1716* were selected to be tagged by *gfp* to observe their dynamics at the protein level during infection. Gene expression profiles showed upregulation during infection. We retained the promoter sequence of each operon in order not to disrupt their regulation on the transcript level. Fusions were performed at the C-terminal position in order to minimize disturbance of protein function. Gene fusion was performed based on isothermal overlapping assembly(6), effectively stitching together three DNA fragments with 40 nucleotide overlaps. The DNA fragments are (i) ~1000 bps upstream of site of fusion, including the gene of interest; (ii) a nine amino-acid linker, the *gfp* sequence and the spectinomycin resistance marker under its own promoter; (iii) ~1000 bps downstream of the fusion site. Assembled fragments were transformed into an unencapsulated (*∆cps2E*) strain expressing *rfp* fused to the highly expressed house-keeping gene, *hlpA*. The *rfp* expression serves as a proxy for the number of pneumococcal cells and basal protein production level. All oligonucleotides involved in generating the fragments are listed in **Table S1**. All pneumococcal strains involved in the study is listed in **Table S2**.

**Infection studies**

*S. pneumoniae* strains were grown until OD_600_ ~0.2, spun down and resuspended in infection medium, RPMI 1640 without phenol red (Life Technologies, NL) supplemented with 1% (v/v) fetal bovine serum (FBS; VWR, NL). In the meantime, a confluent A549 monolayer was rinsed twice with phosphate-buffered saline (PBS, pH 7.4) to remove growth medium. Pneumococcal suspension (multiplicity of infection, MOI ~10, i.e. 10 pneumococcal cells per epithelial cell) in infection medium was added onto the epithelial monolayer. To optimize cell-to-cell contact, soft centrifuging was employed (2000 ×g, 5 min, 4°C), subsequently, the infection culture was incubated in humidified 5% (v/v) CO_2_ atmosphere at 37°C.

To generate heat-inactivated *S. pneumoniae*, we grew the bacteria until the desired OD, spun down and removed supernatant, resuspended the pellet in water and incubated at 56°C for 30 min. This protocol was used to inactivate the bacteria while preserving epitope and other cellular structures(7). To harvest pneumococcal supernatant, *S. pneumoniae* was incubated in infection medium for 60 minutes in a humidified atmosphere (5% CO_2_, 37°C). Further, medium was filtered (0.22 μm) and co-incubated with a confluent monolayer of epithelial cells. To treat the model with resveratrol, resveratrol stock solution was added into the infection medium so that the final concentration was 100 μM. To wash the mucus from the surface of epithelial cells, we removed epithelial growth medium and washed once with warm (37°C) PBS, incubated at 37°C for 30 min in humidified atmosphere and then washed one more time with warm PBS. Cells were then used directly for infection studies with washed epithelial cells. Incubation with mucin in infection medium was done by adding type III porcine mucin into infection medium so that the mucin concentration was 5 g·l^-1^. In addition, the mucin infection medium contains 2 g·l^-1^ glucose.

**Adherence assay**

Confluent A540 monolayers were cultured on 24-wells plates (BD Falcon, VWR, NL). Pneumococcal strains with (D39) and without (*∆cps2E*) capsule were added onto the epithelial monolayer in the same manner as previously described. Five time points were selected to describe the dynamics of adherence, i.e. 0, 30, 60, 120 and 240 mpi (minutes post infection). After the selected time, supernatants were removed and the confluent layers were rinsed twice with PBS (pH 7.4). The rinsed fractions were combined with the supernatant. Detachment solution (Bovine pancreas trypsin, 0.5 mg·ml^-1^, Sigma; 0.5 mM EDTA, Sigma in PBS, pH 7.4) was added to the monolayer, followed by incubation at 37°C for 5 min. The suspension containing A549 and adherent *S. pneumoniae* was pipetted out. Non-adherent and adherent fractions were spun down, diluted with C+Y medium and plated in 2% (v/v) blood Columbia agar. The agar plates were incubated at 37°C. Manual counting of *cfu* (colony forming units) was performed the next day. Afterwards, statistical *t-*tests were performed in an unpaired two-tailed manner.

**qRT-PCR confirmation of host and pathogen genes**

Total host-pathogen RNA was used to confirm RNA-Seq libraries by qRT-PCR. Total RNA was obtained in the same manner as previously described. cDNA was synthesized from total RNA by SuperScript® III Reverse Transcriptase (Life Technologies, NL). The nucleic acids mix contained total RNA sample, random nonamers, dNTP mix (Life Technologies, NL) and RiboLock RNase Inhibitor (Life Technologies, NL). The nucleic acids mix was then incubated at 65°C, 5 min and put directly on ice. First-strand buffer, DTT, RiboLock and Reverse Transcriptase were added into the mix and incubated at 25°C for 5 min, 50°C for 60 min and 70°C for 15 min.

qPCR primers were designed across exons (for host genes) while retaining its species-specificity as confirmed by *in silico* PCR on the opposite species (i.e. host primers to pathogen and *vice versa*). Amplification efficiency for each primer was calculated based on primer ability to double amount of product per cycle. The qPCR mix contained forward and reverse primers, SYBR Green Real-Time PCR Master Mix (Life Technologies, NL) and cDNA – the reactions were on duplicates and two different cDNA concentrations. The PCR temperature scheme was as follows: 95°C, 3 min and then 40 cycles of 95°C, 10 s; 50°C, 30 s and 72°C, 30 s on an iQ™5 Optical System (BioRad, NL). Data were analyzed according to the ∆∆Ct method(13) against housekeeping genes (*ACTB* for the host and *gyrA* for pathogen genes). Gene expression values were then normalized prior to comparison with data generated by sequencing. Fold change was calculated against one sample: the unencapsulated strain at 240 mpi.

**Fluorescence imaging during infection studies**

A549 was cultured as previously described on 8-wells μ-slides (Ibidi, DE). Pneumococcal strains, RA39, RA41, RA42 and RA43, were grown to OD_600_ ~0.2 and added onto the monolayer. Due to lack of capsule, these strains adhere effectively onto the host cells. At different time points, pictures were taken on a DV Elite microscope (Applied Precision) with an sCMOS camera using Solid-State Illumination (Applied Precision) through a 60x oil immersion objective (bright field; 1.42 NA; working distance [WD], 0.15 mm) under humidified 5% (v/v) CO_2_ atmosphere, 37°C.

The images were generated by first focusing on the monolayer of A549 using bright-field microscopy and RFP via the Alexa 594 channel (excitation, 575 nm; emission, 625 nm). Pictures were taken on FITC (excitation, 475 nm; emission, 523 nm) and Alexa 594 channels. Signal strength was quantified by first splitting the image into the three images: bright field, GFP, and RFP. Subsequently, background signals were removed from the GFP and RFP channels by adjusting their thresholds in ImageJ. Arbitrary values (RFP, minimum 200; GFP, minimum 250) were chosen to remove background fluorescence while retaining signals from target genes. This redefinition of threshold converted the channels into binary black and white images. In each channel, the amount of signal was calculated by multiplying the mean signal value by the area. For each image, the ratio of the GFP to RFP signals was calculated. Images were modified for publication using softWoRx, version 6.1 (Applied Precision), and ImageJ.

**Supplemental References**

1. Love MI, Huber W, Anders S. Moderated estimation of fold change and dispersion for RNA-seq data with DESeq2. Genome Biol. 2014;15:550.

2. Wagner GP, Kin K, Lynch VJ. Measurement of mRNA abundance using RNA-seq data: RPKM measure is inconsistent among samples. Theory Biosci Theor Den Biowissenschaften. 2012 Dec;131(4):281–5.

3. Kumar L, E. Futschik M. Mfuzz: A software package for soft clustering of microarray data. Bioinformation. 2007 May 20;2(1):5–7.

4. van Opijnen T, Camilli A. A fine scale phenotype-genotype virulence map of a bacterial pathogen. Genome Res. 2012 Dec;22(12):2541–51.

5. Kjos M, Aprianto R, Fernandes VE, Andrew PW, van Strijp JAG, Nijland R, et al. Bright fluorescent *Streptococcus pneumoniae* for live-cell imaging of host-pathogen interactions. J Bacteriol. 2015 Mar;197(5):807–18.

6. Gibson DG, Young L, Chuang R-Y, Venter JC, Hutchison CA, Smith HO. Enzymatic assembly of DNA molecules up to several hundred kilobases. Nat Methods. 2009 May;6(5):343–5.

7. Hvalbye BKR, Aaberge IS, Løvik M, Haneberg B. Intranasal Immunization with Heat-Inactivated *Streptococcus pneumoniae* Protects Mice against Systemic Pneumococcal Infection. Infect Immun. 1999 Sep;67(9):4320–5.

8. Bolger AM, Lohse M, Usadel B. Trimmomatic: A flexible trimmer for Illumina Sequence Data. Bioinformatics. 2014 Apr 1;btu170.

9. Dobin A, Davis CA, Schlesinger F, Drenkow J, Zaleski C, Jha S, et al. STAR: ultrafast universal RNA-seq aligner. Bioinforma Oxf Engl. 2013 Jan 1;29(1):15–21.

10. Liao Y, Smyth GK, Shi W. featureCounts: an efficient general purpose program for assigning sequence reads to genomic features. Bioinforma Oxf Engl. 2014 Apr 1;30(7):923–30.

11. Huang DW, Sherman BT, Lempicki RA. Systematic and integrative analysis of large gene lists using DAVID bioinformatics resources. Nat Protoc. 2009;4(1):44–57.

12. Huang DW, Sherman BT, Lempicki RA. Bioinformatics enrichment tools: paths toward the comprehensive functional analysis of large gene lists. Nucleic Acids Res. 2009 Jan;37(1):1–13.

13. Livak KJ, Schmittgen TD. Analysis of relative gene expression data using real-time quantitative PCR and the 2(-Delta Delta C(T)) Method. Methods San Diego Calif. 2001 Dec;25(4):402–8.

14. Avery OT, MacLeod CM, McCarty M. Studies on the Chemical Nature of the Substance Inducing Transformation of Pneumoccocal Types. J Exp Med. 1944 Feb 1;79(2):137–58.

| **Table S2.** Primers involved in fragments generation and assembly | |
| --- | --- |
| **Oligonucleotide Name** | **Sequence (5’ → 3’)** |
| 0046_SPD_0475_F | CATCCTATGCGGTGCATGTG |
| 0047_SPD_0475_up_R+hom_GFP | GCAGCTTCTCCACCAGATCCTAAATAATATAGGAGTGGCCAAC |
| 0048_GFP_F+hom_SPD_0475 | GTTGGCCACTCCTATATTATTTAGGATCTGGTGGAGAAGCTGC |
| 0049_GFP_R+hom_SPD_0475 | CTGCTTACCTTTTCATAATTCAATTAGAATGAATATTTCC |
| 0050_SPD_0475_down_F+hom_GFP | GGAAATATTCATTCTAATTGAATTATGAAAAGGTAAGCAG |
| down_0475_R | GTCTCGGAAATGACGCAAAC |
| 0060_SPD_0963_up_F | GGATGGCCCTTATCTGAATG |
| 0061_SPD_0963_up_R+hom_GFP | GCAGCTTCTCCACCAGATCCTAGATTACTGAGGGCACCAATTG |
| 0062_GFP_F+hom_SPD_0963 | CAATTGGTGCCCTCAGTAATCTAGGATCTGGTGGAGAAGCTGC |
| 0063_spec_R+hom_SPD_0963 | CTAAATTTTCTTGAATCTATTCAATTAGAATGAATATTTCC |
| 0064_SPD_0963_down_F+hom_spec | GGAAATATTCATTCTAATTGAATAGATTCAAGAAAATTTAG |
| 0065_SPD_0963_down_R | GACTCTCAGGCATGTCCATC |
| 0068_ssbB_up_F | CCGGACTGGCTCTTGACTTG |
| 0069_ssbB_up_R+hom_GFP | GCAGCTTCTCCACCAGATCCAAATGGCAATTCTTCCTCTTC |
| 0070_GFP_F+hom_ssbB | GAAGAGGAAGAATTGCCATTTGGATCTGGTGGAGAAGCTGC |
| 0071_spec_R+hom_ssbB | CAACTCAGACTTTTTAATTCCAATTAGAATGAATATTTCC |
| 0072_ssbB_down_F+hom_spec | GGAAATATTCATTCTAATTGGAATTAAAAAGTCTGAGTTG |
| 0073_ssbB_down_R | TGGTGATGACACCGTCTTTG |
| SPD_1716_down_R | GCTACCTTGCCTTTACGTTCC |
| 0076_SPD_1716_up_F | CCATCCTTAGACGTCATCTC |
| 0077_SPD_1716_up_R+hom_GFP | GCAGCTTCTCCACCAGATCCCTTGCCATTGTCTTTGACCC |
| 0078_GFP_F+hom_SPD_1716 | GGGTCAAAGACAATGGCAAGGGATCTGGTGGAGAAGCTGC |
| 0079_spec_R+hom_SPD_1716 | CCTGATGAAGCCAGATAATACAATTAGAATGAATATTTCC |
| 0080_SPD_1716_down_F+hom_spec | GGAAATATTCATTCTAATTGTATTATCTGGCTTCATCAGG |

| **Table S3.** *S. pneumoniae* strains involved in the infection studies | | | |
| --- | --- | --- | --- |
| **Strain** | **Capsule** | **Description** | **Ref** |
| D39 | Present | pathogenic, serotype 2 | Avery *et al*(14) |
| *∆cps2E* | Absent | D39 *∆cps2E*::Kanʳ | Kjos *et al*(5) |
| RA39 | Absent | D39 *∆cps2E*::Kanʳ *hlpA_hlpA-rfp*_Camʳ *SPD0392_gfp*::Spcʳ | This study |
| RA41 | Absent | D39 *∆cps2E*::Kanʳ *hlpA_hlpA-rfp*_Camʳ *SPD0475_gfp*::Spcʳ | This study |
| RA42 | Absent | D39 *∆cps2E*::Kanʳ *hlpA_hlpA-rfp*_Camʳ *SPD1711_gfp*::Spcʳ | This study |
| RA43 | Absent | D39 *∆cps2E*::Kanʳ *hlpA_hlpA-rfp*_Camʳ *SPD1716_gfp*::Spcʳ | This study |

**Tables S1: Clusters of Pneumococcal Genes, 4: Epithelial Differential Gene Expression**, **5: Pneumococcal Differential Gene Expression** and **6: Clusters of Epithelial Immunity and Apoptotic Genes** are available as a separate Excel file **(Additional file 2).**
